# Novel Sequence Features of DNA Repair Genes/Proteins from *Deinococcus* Species Implicated in Protection from Oxidatively Generated Damage

**DOI:** 10.3390/genes9030149

**Published:** 2018-03-08

**Authors:** F. M. Nazmul Hassan, Radhey S. Gupta

**Affiliations:** Department of Biochemistry and Biomedical Sciences, McMaster University, Hamilton, ON L8N 3Z5, Canada; hassaf5@mcmaster.ca

**Keywords:** *Deinococcus* species, radiation and desiccation resistance, conserved signature indels, oxidatively generated damage, CXXC motifs in protein sequences, DsbA proteins, DsbB proteins, UvrA1 proteins, UvsE proteins

## Abstract

*Deinococcus* species display a high degree of resistance to radiation and desiccation due to their ability to protect critical proteome from oxidatively generated damage; however, the underlying mechanisms are not understood. Comparative analysis of DNA repair proteins reported here has identified 22 conserved signature indels (CSIs) in the proteins UvrA1, UvrC, UvrD, UvsE, MutY, MutM, Nth, RecA, RecD, RecG, RecQ, RecR, RuvC, RadA, PolA, DnaE, LigA, GyrA and GyrB, that are uniquely shared by all/most *Deinococcus* homologs. Of these CSIs, a 30 amino acid surface-exposed insert in the *Deinococcus* UvrA1, which distinguishes it from all other UvrA homologs, is of much interest. The *uvrA1* gene in *Deinococcus* also exhibits specific genetic linkage (predicted operonic arrangement) to genes for three other proteins including a novel *Deinococcus*-specific transmembrane protein (designated dCSP-1) and the proteins DsbA and DsbB, playing central roles in protein disulfide bond formation by oxidation-reduction of CXXC (C represents cysteine, X any other amino acid) motifs. The CXXC motifs provide important targets for oxidation damage and they are present in many DNA repair proteins including five in UvrA, which are part of Zinc-finger elements. A conserved insert specific for *Deinococcus* is also present in the DsbA protein. Additionally, the *uvsE* gene in *Deinococcus* also shows specific linkage to the gene for a membrane-associated protein. To account for these novel observations, a model is proposed where specific interaction of the *Deinococcus* UvrA1 protein with membrane-bound dCSP-1 enables the UvrA1 to receive electrons from DsbA-DsbB oxido-reductase machinery to ameliorate oxidation damage in the UvrA1 protein.

## 1. Introduction

Members of the genus *Deinococcus*, which are part of the phylum Deinococcus-Thermus [1,2,3,4,5,6] are characterized by their extraordinary tolerance to ionizing radiation (IR), ultraviolet radiation (UV) and desiccating conditions [7,8,9,10]. Due to their high level of resistance to radiation and desiccation, which are lethal or growth inhibitory to most other organisms, *Deinococcus* species have been extensively studied for understanding the biochemical mechanisms responsible for their resistance characteristics [7,9,11,12,13,14,15,16,17,18]. Earlier work shows that radiation (ionizing or UV) or desiccating conditions produce comparable DNA damage in *Deinococcus* species as in sensitive organisms (e.g., *Escherichia coli*) but in contrast to the sensitive organisms, damaged DNA in *Deinococcus* species gets efficiently repaired leading to their observed resistance phenotype [9,11,12,14,19,20]. Similar to other prokaryotic organisms, *Deinococcus* species possess classical DNA repair machinery consisting of the base excision repair (BER), nucleotide excision repair (NER), mismatch repair (MMR) and double-strand break (DSB) repair pathways [11,12,16,21]. Most of the proteins in these pathways are conserved and thus far very few novel aspects of these proteins or DNA repair pathways have been identified that could account for the highly efficient mode of DNA repair in *Deinococcus* species [11,12,16,21]. The genomes of *Deinococcus* species also contain a number of proteins which are specific for this group of bacteria [21,22] and the genes for some of them (e.g., DdrA, DdrB, DdrD, PprA) are induced upon radiation or desiccation exposure [23,24], suggesting that they play important roles in DNA damage response [11,12,14,23]. However, our current understanding of the cellular functions of these proteins, or other proteins involved in DNA repair processes, does not satisfactorily account for the efficient repair of DNA observed in *Deinococcus* species [11,12,25,26,27].

The detrimental effects of radiation or desiccation on living organisms, including DNA damage and strand breaks, are mainly produced by oxidatively generated damage caused by reactive oxygen species (ROS) [9,12,14,19,20,28,29]. However, the damaging effects of ROS are not limited to DNA but oxidation and inactivation of proteins are also important consequences [12,14,20,29,30,31]. An important observation in this context is that while DNA from both radiation-sensitive and -resistant organisms is equally susceptible to radiation or desiccation-induced damage, there is much less damage of the proteome in resistant organisms such as *Deinococcus* in comparison to sensitive organisms [9,12,14,19,20]. This observation indicates that the ability of *Deinococcus* species to withstand the effects of high doses of radiation and other ROS-inducing conditions is in large part due to their unique ability to protect their proteome from oxidatively generated damage [9,12,14,20]. Although *Deinococcus* species contain several proteins involved in antioxidant defense including thioredoxin, glutaredoxin, thioredoxin reductase, glutathione, glutathione reductase, etc., their presence is not unique to these bacteria [12]. However, recent studies show that *Deinococcus* species contain much higher intracellular concentration of Mn^+2^ and Mn^+2^-phosphate complexes, which are potent scavengers of superoxide radicals, suggesting that the high Mn^+2^/Fe^+2^ ratio in these organisms could provide protection from oxidative stress [12,14,20,32,33,34,35]. While the role played by high Mn^+2^/Fe^+2^ concentrations in protecting from oxidative stress is of importance, it does not explain the selective protection of proteome (as compared to DNA) in *Deinococcus* species. Thus, it is possible that in addition to the role played by Mn^+2^ complexes and other small molecules, proteins involved in DNA repair processes from *Deinococcus* species may contain certain novel molecular attributes that protect them from oxidatively generated damage. However, thus far no unique features in DNA repair proteins from *Deinococcus* have been identified.

Our comparative genomic analyses on members of the phylum Deinococcus-Thermus have identified large numbers of novel sequence features that are specific for the *Deinococcus* species [5,22,36]. These novel sequence characteristics include several conserved signature indels (CSIs) in important proteins of *Deinococcus* species as well as more than 28 conserved signature proteins (CSPs), whose homologs are only present in all/most *Deinococcus* species [5,22]. Earlier work on CSIs shows that they are generally present on protein surfaces and mediate novel protein-protein or protein-DNA interactions, which are important for the CSI-containing organisms [37,38,39,40]. Similarly, the CSPs found only in a given group of organisms, are also predicted to play important cellular functions in these organisms [41,42,43]. A number of such proteins (viz. DdrB, PprA), which are specifically found in species from either the genus *Deinococcus* or the order Deinococcales have been extensively studied and are known to play important role in the DNA repair process [26,27,44,45,46]. In view of the important roles that CSIs and CSPs play in conferring novel cellular functions, the present study was undertaken to identify CSIs which are specifically found in the DNA repair proteins from *Deinococccus* species. Results of these studies have identified 22 CSIs in many important DNA repair proteins from different pathways. Of the identified CSIs, one CSI of much interest consists of a 30 amino acid insert uniquely found in all of the UvrA1 homologs from *Deinococcus* species. The UvrA1 protein, which is part of the nucleotide excision repair (NER) pathway [12,47], plays a central role in the DNA repair process due to its ability to initially recognize a broad range of DNA damages including cyclobutane pyrimidine dimers and 6,4-photoproducts formed by UV light and multiple other types of damaged nucleotides/bases resulting from exposure to ionizing radiation [11,12,47,48,49]. Subsequently, other proteins in the NER pathway viz. UvrB and UvrC excise the damaged region and the gap created is filled by DNA polymerase I and ligated by DNA ligase [11,12,47,48,49]. Our studies show that the gene for UvrA1 in *Deinococcus* species exhibits a novel genetic organization (i.e., operonic arrangement) with the genes for a *Deinococcus*-specific CSP (dCSP-1, predicted to be a transmembrane protein) and two other membrane-associated proteins (DsbA and DsbB), which are known to play central roles in disulfide bond formation in proteins by oxidation-reduction of cysteine residues in CXXC motifs [50,51,52]. Our analysis shows that many DNA repair proteins contain surface exposed CXXC motifs, which are highly susceptible to oxidation damage [30,53,54] and of these UvrA protein contains 5 CXXC motifs, which are part of three zinc-finger elements commonly utilized by proteins for recognizing either specific regions in DNA or for mediating protein-protein interactions [47,54,55,56]. Additionally, our results also reveal that the UvsE protein, central to the UvsE-dependent pathway of excision DNA repair [49], also contains a 1 amino acid CSI specific for *Deinococcus* homologs and the gene for this protein exhibits a novel genetic linkage with the gene for a predicted transmembrane protein. The significances of these findings as well as a number of other novel observations on DNA repair proteins from *Deinococcus* species regarding their ability to protect their DNA repair machinery from oxidatively generated damage are discussed.

## 2. Materials and Methods

### 2.1. Identification of CSIs in DNA Repair Proteins

Identification of conserved signature indels in DNA repair proteins was carried out as described in our earlier work [5,57]. In brief, BLASTp [58] searches were conducted on different DNA repair proteins from the genome of *Deinococcus radiodurans* R1 [59] to retrieve homologs from different members of the Deinococcus-Thermus phylum and representative members from other groups of bacteria. Multiple sequence alignments of different proteins were created using ClustalX [60]. The alignments were visually inspected to identify any conserved insertion or deletion, which was specific for members of the genus *Deinococcus* and which was flanked on both sides by at least 5–6 conserved amino acids in the neighboring 30–40 amino acids. The specificities of these indels for *Deinococcus* species were evaluated by performing additional BLASTp searches on short sequence segments containing the insertions or deletions and their flanking conserved regions (60–100 amino acids long). SIG_CREATE and SIG_STYLE (available on Gleans.net) were then used to create the formatted signature files showing the presence of CSIs in the sequence alignments [57]. Although sequence information is shown for only a limited number of species in these alignments, unless otherwise indicated, all of the CSIs shown here are specific for the *Deinococcus* species and similar CSIs were not detected in any other organism in the top 250 BLAST hits analyzed.

### 2.2. Protein-Protein Interactions (PPIs) and Genetic Neighborhood Analyses

The STRING database was initially used to determine any unique association observed for the DNA repair proteins from *Deinococcus* species. The STRING database contains information regarding protein-protein interactions (PPIs) based on experimental data and it also predicts interactions based on co-occurrence of the proteins, gene fusion data, co-expression of the proteins and genetic neighborhood analysis [61,62]. More detailed genetic-linkage analysis on the genes for selected proteins (e.g., UvrA1 and UvsE) was carried out by examining the position of a given gene and its neighboring genes from the graphic views of the genomes. Intergenic distances and the direction of transcription for different genes were also determined based on the genome sequences.

### 2.3. Homology Modeling of the Uvra1 Protein and Other Proteins from Deinococcus Species

Three-dimensional structure models of the UvrA1, UvrC and UvsE proteins from *D. radiodurans* were developed from the full-length sequences of these proteins using the homology modeling technique [63]. The secondary structure analysis on the sequences of these proteins from *D. radiodurans* was initially performed via PSIPRED v3.3 web server [64]. Suitable templates for homology modeling were identified by using PSI-Blast search [58] against the Protein Data Bank (PDB) [65]. The templates used for construction of homology models of the UvrA, UvrC and UvsE proteins of *D. radiodurans* were based on the following structures; UvrA1, *Geobacillus* sp. Y412MC52 (PDB id: 3UWX) [66]; UvrC, *Thermotoga maritime* (PDB id: 2NRT) [67]; and UvsE, *Sulfolobus acidocaldarius* (PDB id: 3TC3) [68]. Based on these template structures, 200 models were initially generated using Modeller v9.14 [63] and ranked using discrete optimized protein energy (DOPE) potential scores [69]. The ModRefiner program was then used for the refinement of selected models [70]. The secondary structure elements in the regions containing CSIs were examined and compared with results of the PSIPRED analysis to ensure their reliability. The assessment of the final structure models was conducted using five independent servers: PROSA [71,72], RAMPAGE [73], ERRAT [74], Verify3D [75] and QMEAN [76]. All the modeled structures were visualized and analyzed using the molecular visualization program PyMol (http://www.pymol.org).

## 3. Results

### 3.1. Conserved Signature Indels in DNA Repair Proteins Specific for the Deinococcus Species

To explore whether the proteins involved in DNA repair pathways in *Deinococcus* species contain any unique sequence features differentiating them from homologs found in other prokaryotic organisms, multiple sequence alignments of various DNA repair proteins from *Deinococcus* and other representative groups of bacteria were created. These alignments were examined for the presence of any conserved signature indels (CSIs) that are specifically found in the *Deinococcus* homologs [5,36,37,38,39,40,41,42,43,44,45,46,47,48,49,50,51,52,53,54,55,56,57]. These analyses have identified 22 novel CSIs in 20 different DNA repair related proteins which, except for an isolated exception, are uniquely found in the *Deinococcus* homologs. A summary of the identified *Deinococcus*-specific CSIs in different DNA repair proteins is provided in Table 1. Of these CSIs, two CSIs in the DNA repair protein RadA, one of which is specific for the Deinococcus-Thermus phylum and the other for the order Deinococcales, were described in our recent work [5]. As seen from Table 1, the CSIs distinguishing the *Deinococcus* homologs from other bacteria are present in large numbers of DNA repair proteins that are part of different pathways [11,12]. The proteins containing the CSIs include UvrA1 and UvrC from the NER pathway [18,47]; UvsE protein from the UV damage endonuclease-dependent excision repair (UVER) pathway [49,50,51,52,53,54,55,56,57,58,59,60,61,62,63,64,65,66,67,68,69,70,71,72,73,74,75,76,77]; MutY, MutM and Nth proteins from the base-excision repair (BER) pathway [11,12]; RecA, RecD, RecG, RecQ, RecR, RuvC and RadA proteins from the homologous recombination (HR) pathway [11,12] and the proteins PolA, DnaE, LigA, UvrD, GyrA and GyrB which play central roles in multiple DNA repair pathways [11,12].

**Figure 1:**
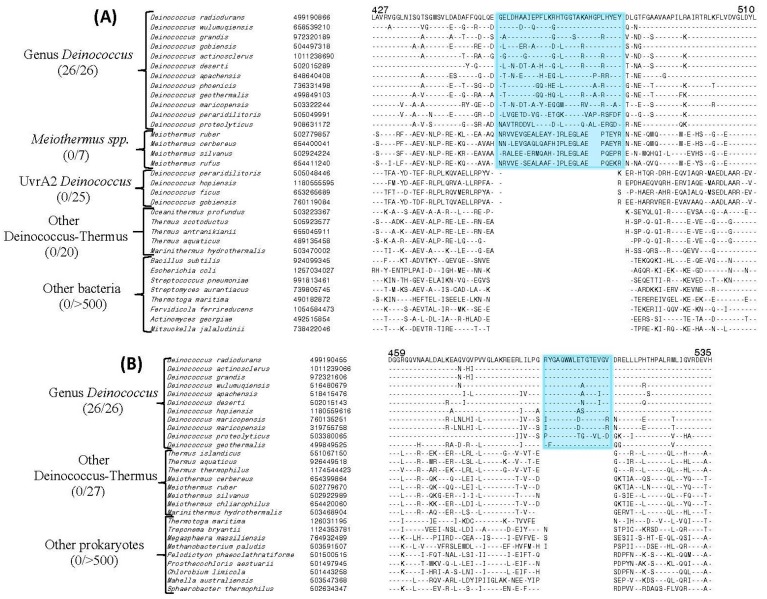
Conserved signature indels in the UvrA1 and UvrC proteins that are specific for the *Deinococcus* homologs. (**A**) Partial sequence alignment of the UvrA protein showing a 30 amino acid insertion in a conserved region that is uniquely shared by the UvrA1 homologs from all 26 genome-sequenced *Deinococcus* species including *Deinococcus aerius.* This insert is not shared by the UvrA2 homologs of *Deinococcus* spp. but a shorter unrelated insert in this position is present in *Meiothermus* spp.; (**B**) Excerpts from the sequence alignment of UvrC protein showing a 16 amino acid insertion that is specific for *Deinococcus* homologs. The dashes (–) in these as well as other sequence alignments indicate identity with the amino acid present on the top line. The numbers on the top indicate the location of the sequence in the *Deinococcus radiodurans* protein.

**Figure 2:**
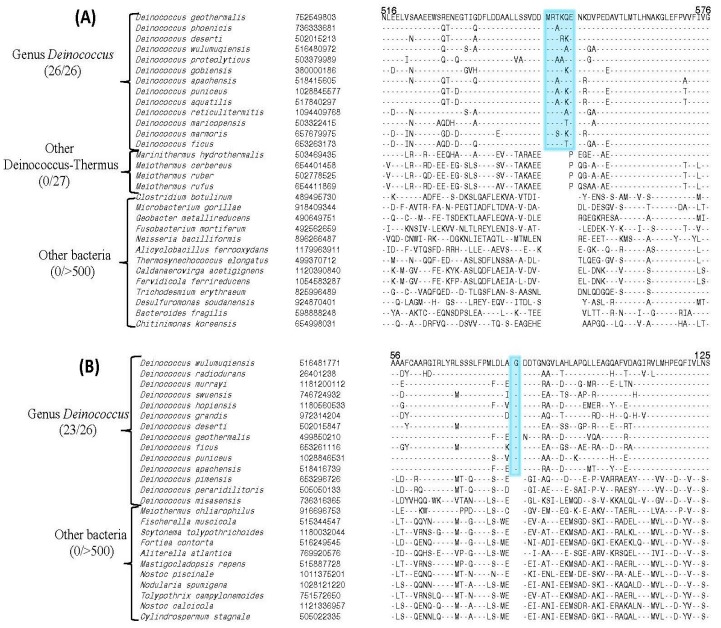
Partial sequence alignment showing conserved indels in (**A**) DNA helicase (UvrD) protein and (**B**) UV damage endonuclease (UvsE) protein which are specific for *Deinococcus*.

Of the identified CSIs, sequence information for two prominent conserved inserts found in the proteins UvrA1 and UvrC is provided in Figure 1. As seen from Figure 1A, the UvrA1 protein from *Deinococcus* contains a 30 amino acid insertion within a conserved region that is uniquely shared by all *Deinococcus* species. The UvrA homologs from other groups of bacteria as well as the UvrA2 homologs from *Deinococcus* species lack this large insert, indicating that this insert is specific for the UvrA1 homologs. Although a shorter insert in this position is present in the *Meiothermus* species, this insert shows minimal sequence similarity to the insert in the *Deinococcus* homologs indicating that it has very likely originated independently. In addition to the UvrA1 protein, UvrC protein from *Deinococcus* species also contains a 16 amino acid insertion in a conserved region that distinguishes the *Deinococcus* homologs from the UvrC homologs in all other bacteria (Figure 1B). Sequence information for two other *Deinococcus*-specific CSIs found in the UvrD and UvsE proteins are presented in Figure 2. Sequence information for the remainder of the *Deinococcus*-specific CSIs in DNA repair proteins identified in this work is provided in Figures S1–S15. Although sequence information for different CSIs is shown here for a limited number of species, unless indicated otherwise, these CSIs are specifically present in all genome-sequenced *Deinococcus* species. Due to the specificities of these CSIs for the *Deinococcus* species, the genetic changes responsible for most of these CSIs likely occurred in a common ancestor of the genus *Deinococcus*.

### 3.2. Locations of the Conserved Indels (CSIs) in the Structures of the Proteins

The locations of the identified CSIs in the structures of a number of DNA repair proteins viz. UvrA1, UvrC, UvrD and UvsE, were examined. Of these four proteins, three dimensional structure of UvrD protein is available from *D. radiodurans* [78]. For the other three proteins, three-dimensional structural models for the *D. radiodurans* homologs were constructed by the homology modeling approach using suitable available template structures as described in the Methods section [63].

**Figure 3:**
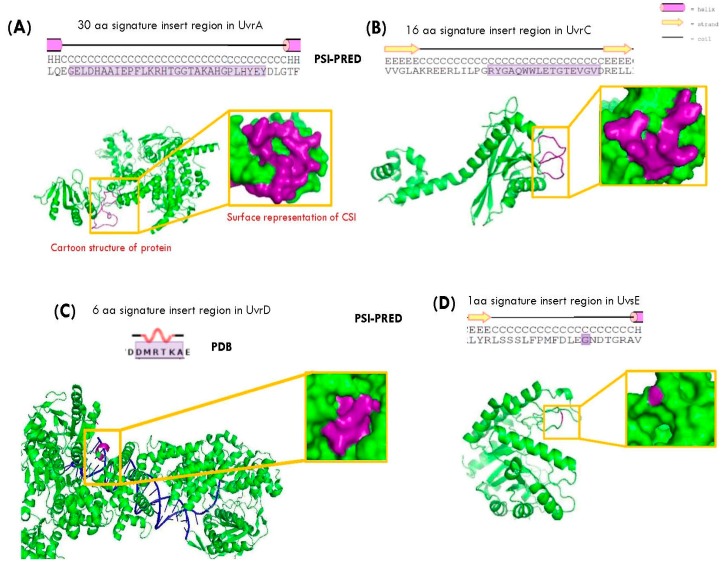
Secondary structure characteristics and structural locations of the identified CSIs in some DNA repair proteins. (**A**) Carton and surface representation of the location of the 30 amino acid CSI in the modeled structure of the UvrA1 protein from *D. radiodurans*; (**B**) Location of the 16 amino acid CSI in the modeled structure of UvrC protein; (**C**) Cartoon and surface representation of a 6 amino acid CSI in crystalized UvrD protein (PDB id: 4C2T); (**D**) Structural analysis of the 1 amino acid CSI in UvsE protein in the modeled structure of *D. radiodurans*.

All of the generated models were of good stereo-chemical qualities as assessed by means of five independent structural validation servers (see Methods section). The locations (surface representations) of the identified CSIs in the structures of the modeled or solved structures of the proteins UvrA1, UvrC, UvrD and UvsE are shown in Figure 3. In addition, this figure also presents information regarding the secondary structures of the CSI-containing region (shown on top in purple color) as predicted by the PSIPRED server [64]. As seen from Figure 3, the CSIs in the UvrA1, UvrC and UvsE proteins are present in surface exposed loops of the modeled proteins. The 6 amino acid CSI in the UvrD protein is also located on the protein surface but it is present within an alpha helix, which appears to play a role in stabilizing the binding of the adjacent loop with the single-stranded DNA [78].

### 3.3. Novel Genomic Organization-Linkage of the Genes for UvrA1 and UvsE Proteins in Deinococcus Species

**Figure 4:**
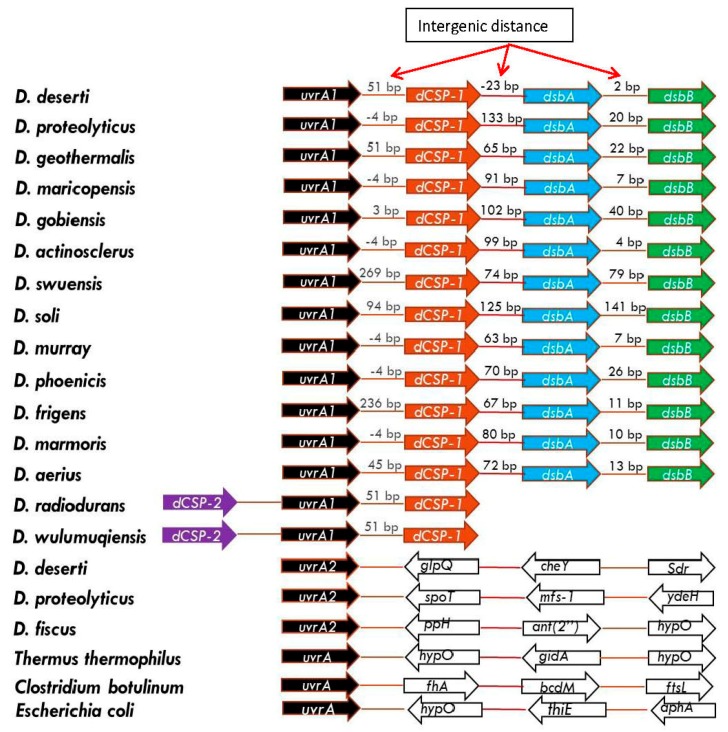
Genomic neighborhood of the gene for UvrA1 protein from *Deinococcus* species showing its specific linkage to the genes for dCSP-1, DsbA and DsbB proteins. The genes for these four proteins are oriented in the same direction and their intergenic distances in most cases are <100 base pairs indicating that they likely form an operon. The genes for UvrA2 from *Deinococcus* as well as the *uvrA* genes from other bacterial groups do not show specific genetic linkage to genes for any of these proteins. In *D. radiodurans* and *D. wulumuqiensis*, the gene for UvrA1 shows specific genetic linkage to the genes for dCSP-2 and dCSP-1 proteins, both of which are *Deinococcus*-specific.

The CSIs in most studied proteins are located in surface loops and commonly involved in facilitating novel protein-protein or protein-ligand interactions [37,38,39,40]. To determine, whether any of the CSI-harboring proteins from *Deinococcus* exhibit any novel interactions, the interaction profiles of different CSI-containing proteins was investigated using the STRING database [61]. This database predicts protein-protein interactions (PPIs) based on experimental data as well as co-occurrence of the proteins, gene fusion data, co-expression of the proteins and genetic neighborhood analysis [61,62]. Of the different DNA repair proteins containing the CSIs, the STRING server predicted novel protein-protein interactions for two proteins. In the first case, UvrA1 protein from *Deinococcus* was found to exhibit unique interactions with three other proteins based on its conserved genomic neighborhood. The three proteins whose genes were found to be in the immediate neighborhood of the *uvrA1* gene from *Deinococcus* included a conserved *Deinococcus*-specific CSP, which is referred to here as dCSP-1 (for *Deinococcus-*specific conserved signature protein-1; accession number NP_295493) and two other proteins DsbA and DsbB, which are known to play central roles in disulfide bond formation in proteins [50,51,52]. In the STRING database, information regarding PPIs was present for only five *Deinococcus* species (*D. radiodurans*, *Deinococcus geothermalis*, *Deinococcus deserti*, *Deinococcus proteolyticus* and *Deinococcus maricopensis*) and of these four species, all except *D. radiodurans* showed a genetic linkage of the *uvrA1* to the genes for the above three proteins (Figure 4). In case of *D. radiodurans*, only the gene for the dCSP-1 protein was indicated to be in the immediate neighborhood of the *uvrA1* gene. In contrast to the *Deinococcus* species, no other bacterial species exhibited any genetic linkage of the *uvrA* gene to the genes for any of these proteins.

The close genetic linkage of the *uvrA1* gene in *Deinococcus* species to the genes for the above three proteins by STRING analysis prompted us to examine in detail the genomic neighborhood of the *uvrA1* gene in all available *Deinococcus* genomes. For these studies, the genomic neighborhood of the *uvrA1* gene was manually examined in different *Deinococcus* genomes and a summary of the results of these analyses is presented in Figure 4. These studies revealed that of the 26 *Deinococcus* genomes currently available, 24 of them exhibited identical genomic organization where the genes for dCSP-1, DsbA and DsbB proteins were present in the immediate neighborhood of the *uvrA1* gene and their relative gene orders as well as orientations were identical (Figure 4). In the remaining two genomes, corresponding to *D. radiodurans* and *D. wulumuqiensis*, only the gene for the dCSP-1 was found immediately downstream of the *uvrA1* gene but the linkage to the genes for DsbA and DsbB proteins was not observed (Figure 4). However, in these two species, the gene for another novel CSP referred to here as dCSP-2 (Accession number WP_010888407.1), which is only found in *D. radiodurans* and *D. wulumuqiensis*, was located immediately upstream of the *uvrA1* gene. The indicated novel genomic arrangements were only observed for the *uvrA1* gene from *Deinococcus* species and similar gene arrangement was not found in any other studied bacteria (Figure 4 and other results not shown). As noted earlier, *Deinococcus* species contain another homolog of the UvrA protein (UvrA2) [79,80] and the gene for this homolog also exhibited no linkage to these genes (Figure 4). In all of these cases, the genes in the neighborhood of *uvrA* were found to be different and showed no specific pattern (Figure 4).

In prokaryotic organisms, ~60% of the genes are present in polycistronic operons [81,82]. An operon consists of a cluster of genes arranged in tandem on the same strand of a genome sharing common promoter and terminator. The specific linkage of the genes for UvrA1, dCSP-1, DsbA and DsbB proteins in most *Deinococcus* species and of the genes for dCSP-2, UvrA1 and dCSP-1 in *D. radiodurans* and *D. wulumuqiensis*, suggests that these two sets of genes likely form distinct operons. This inference is consistent with the observation that all of these genes are present on the same strand of DNA and they are transcribed in the same direction. We have also measured the genetic distances between these genes in different *Deinococcus* genomes and in most cases, the intergenic distances separating these genes are <100 base pairs (bp) (Figure 4). Analyses of genes from well-studied prokaryotic species indicate that when the genetic distance between two gene exhibiting similar phylogenetic profiles is <200 bp, there is a strong likelihood that these genes are part of an operon [82]. We also used the DOOR 2.0 database to determine whether the genes for these four proteins are part of an operon [83]. The DOOR 2.0 database contains computationally predicted operons of prokaryotic genomes and its accuracy for correctly predicting the operonic organization for *Bacillus subtilis* and *E. coli* is >90% [83]. Information for six *Deinococcus* species was available in the DOOR 2.0 database and based on its computational prediction, the genes for UvrA1, dCSP-1, DsbA and DsbB proteins were present in a single operon in 3 out of 6 species (viz. *D. deserti*, *D. gobiensis* and *D. geothermalis*). Of the remaining three species, in *D. proteolyticus* and *D. maricopensis*, the genes for UvrA1 and dCSP-1 proteins were indicated to be part of one operon, whereas the genes for DsbA and DsbB proteins were part of an adjacent operon. However, since the intergenic distance between these two neighboring sets of genes (or operons) is <100 bp, it is highly likely that all four of these genes are part of a single operon. In *D. radiodurans*, an operonic arrangement was observed only for the genes for dCSP-2, UvrA1 and dCSP-1 proteins and the genes for DsbA and DsbB were not present in its neighborhood [82,83].

**Figure 5:**
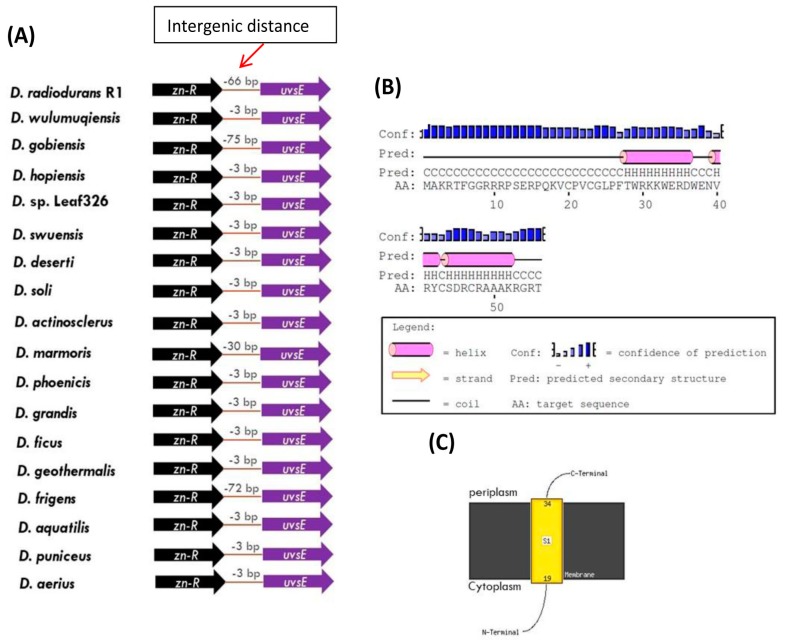
(**A**) Genomic neighborhood analysis of the gene for UvsE protein showing its specific genetic linkage in *Deinococcus* species to the gene for Zn-ribbon protein (Zn-R). The genes for these two proteins are oriented in the same direction and their coding regions overlap suggesting that they form an operon. (**B** and **C**) Predicted secondary structure and membrane topology of the Zn-ribbon protein (Accession ID: AFD24462.1).

Besides the UvrA1 protein, our genetic neighborhood analysis also reveals that the gene for the UvsE protein in *Deinococcus* species also exhibits a novel and specific genetic linkage to the gene for a Zn-ribbon (Zn-R) protein (accession number AFD24462.1) (Figure 5). The DOOR.2 database also predicts that the genes for UvsE and Zn-R are part of an operon in *Deinococcus* species.

### 3.4. Structural and Biochemical Characteristics of the Proteins Linked to the UvrA1 Protein

**Figure A1:**
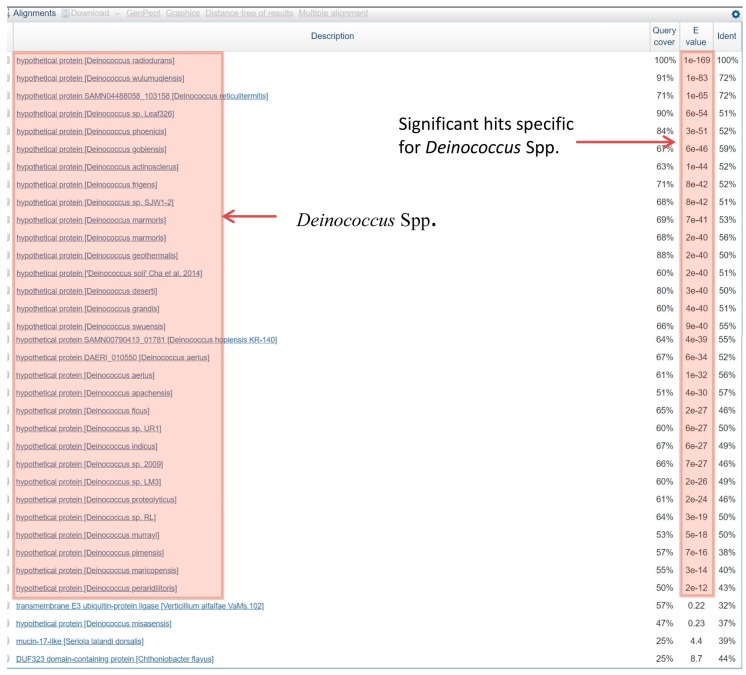
Blast Search Results Showing the *Deinococcus* specificity of the Protein dCSP-1.

Our results indicate that the genes for UvrA1, dCSP-1, DsbA and DsbB proteins in *Deinococcus* species exhibit specific genetic linkage and they likely form an operon. As the genes within an operon generally carry out related functions [82,84,85], it is of much interest to understand the functions of the three proteins that are genetically linked to the UvrA1 protein. Of these three proteins, dCSP-1 is a protein that is uniquely found in *Deinococcus* species. In our earlier work, this protein was identified as a CSP that was specifically found in all *Deinococcus* species for which genome sequence information was available [5,22]. The specificity of this protein for *Deinococcus* species was re-examined by BLASTp searches and the results obtained confirm that this protein is a distinctive characteristic of all *Deinococcus* species (Appendix A, Figure A1).

**Figure 6:**
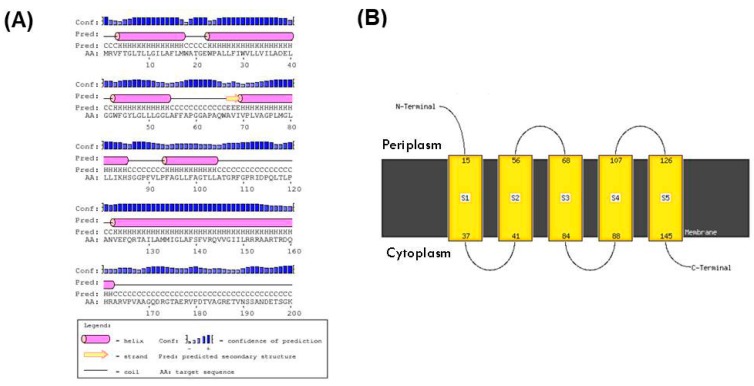
Secondary structure prediction (**A**) and membrane topology (**B**) of the dCSP-1 protein (Accession ID: NP_295493). Most residues in this protein are present in helix regions and it is predicted to be a cytoplasmic membrane protein with five transmembrane helices.

The protein dCSP-1 (NP_295493) is 247 amino acids long in *D. radiodurans* and it is annotated as a hypothetical protein of unknown function. To gain insights into the possible function of this protein, its sequence was analyzed using the PSIPRED server [64]. This server uses multiple methods for predicting secondary structure of proteins and it also indicates whether a given protein is a membrane protein and its membrane topology [64]. The secondary structure predicted for the dCSP-1 by the PSIPRED server is shown in Figure 6A. Most of the residues from dCSP-1 are present in 6 alpha helices (shown in magenta color) and it contains only a small beta strand region shown in yellow. Based on its predominantly helical structure, the PSIPRED server predicts that dCSP-1 is a transmembrane protein containing five membrane-spanning regions and its overall membrane topology is as shown in Figure 6B.

**Figure 7:**
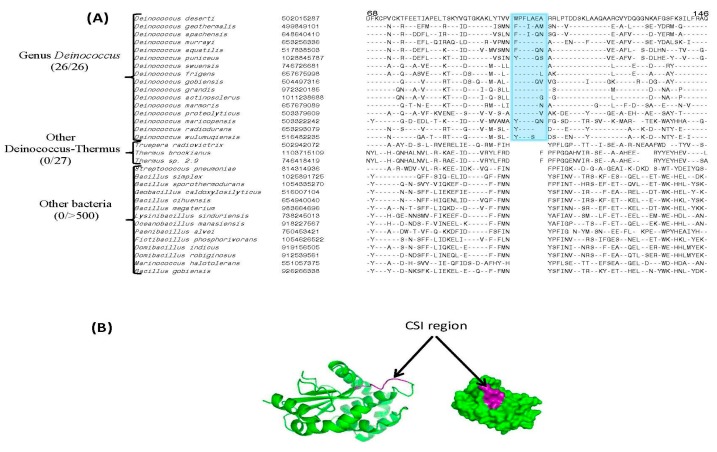
(**A**) Partial sequence alignment of the DsbA oxidoreductase protein showing a 5–7 amino acid insertion in a conserved region that is specific for *Deinococcus* species; (**B**) Location of the CSI in the modeled structure of DsbA protein from *D. deserti* constructed using the template structure of DsbA protein from *Bacillus subtilis* (PDB id: 3eu3).

The other two proteins DsbA and DsbB, showing genetic linkage to the UvrA1 and dCSP-1 proteins function together in the formation of disulfide bonds in proteins [50,51]. Of these, DsbA is localized in the periplasmic space and it catalyzes intrachain disulfide bond formation in newly formed proteins as they emerge in the periplasm. The continued functioning of DsbA requires DsbB, which is a cytoplasmic membrane protein containing two CXXC motifs, which oxidizes DsbA to regenerate its active form [50,51,86]. We have examined whether the proteins DsbA or DsbB contain any novel sequence features that are specific for the *Deinococcus* species. These studies have identified a 5–7 amino acid insert in a highly conserved region of the DsbA protein that is specifically present in all *Deinococcus*-species (Figure 7). While all other *Deinococcus* species contain a 7 amino acid insertion in the DsbA, *D. radiodurans* and *D. wulumuqiensis* contain a shorter 5 amino acid insert in the same position (Figure 7).

As noted above, the gene for the UvsE protein also exhibits a specific genetic linkage to the gene for a Zn-ribbon (Zn-R) protein in *Deinococcus* species (Figure 5A). The genes for these two proteins show partial overlap in most *Deinococcus* species (Figure 5A). The Zn-ribbon protein linked to UvsE is 67 aa long and analysis of its sequence by the PSIPRED server indicates that this protein also contains a transmembrane helix (Figure 5B) and is predicted to be a cytoplasmic membrane protein (Figure 5C).

### 3.5. Presence of CXXC Motifs in DNA Repair Proteins

**Figure 8:**
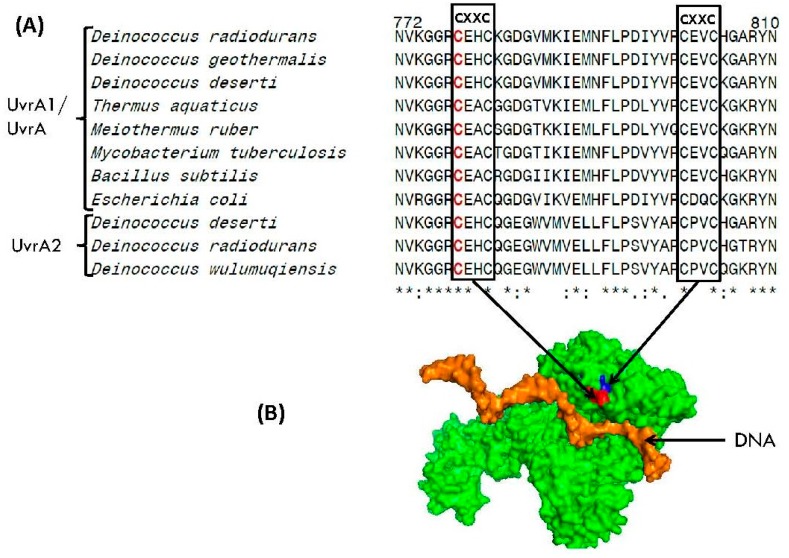
(**A**) Partial sequence alignment of the C-terminal region from UvrA protein showing the presence of a Zinc-finger element containing CXXC motifs. The CXXC motifs shown here are conserved in all UvrA homologs. Mutational studies on the cysteine residue marked in red indicates that it plays an important role in the functioning of the UvrA protein [54,56]; (**B**) Location of two the CXXC motifs, which are part of a Zinc finger element, in the structure of UvrA protein.

The observed genetic linkage of UvrA1 to the DsbA and DsbB proteins, which provide the main cellular machinery for oxidation-reduction of CXXC motifs in proteins, indicates that this aspect should be of importance for *Deinococcus* species. Hence, we have examined the sequences of various DNA repair proteins for the presence of CXXC motifs. The results of our analysis indicate that CXXC motifs are present in a large number of DNA repair proteins including UvrA1, DNA ligase, DNA polymerase II subunit gamma/tau, MutY, MutM, Nth, Rad 25, RecO, RecR, RecQ, SbcC and RadA (Appendix A Table A1 and Figure S17(A–L)). While all other DNA repair proteins listed in Table A1 contain either one or two CXXC motifs, the UvrA1 protein is found to contain five CXXC motifs, indicating that these motifs should play important role in the functioning of this protein. All of the CXXC motifs in UvrA1 are located on protein surface and they are parts of three zinc finger elements [47,55], commonly utilized by proteins for binding to specific regions in DNA or for mediating protein-protein interactions [55,56,66,79,87,88]. Partial sequence alignment of the UvrA protein showing two of the CXXC motifs, which are present near the C-terminal end, as well as the locations of these motifs in the structure of UvrA are shown in Figure 8. Earlier work has shown that substitution of one of the cysteine (marked in red) from these CXXC motif causes functional inactivation of the UvrA1 protein [54]. 

## 4. Discussion

*Deinococcus* species are highly resistant to UV and ionizing radiations and prolonged desiccation, due to their ability to protect their proteome from the harmful effects of ROS [12,14,19,20,28,89]. However, the biochemical mechanisms enabling these bacteria to protect their critical proteome from oxidatively-generated damage remain unidentified [12]. In this context, the results of our comparative analyses of DNA repair proteins, which have identified multiple highly-specific molecular signatures in the forms of CSIs that are specific for *Deinococcus* homologs, are of much interest. Earlier work on CSIs provides evidence that the genetic changes of this kind play important functional roles in the organisms for which they are specific [38,39,90]. Further, most of the studied CSIs in proteins, including in the DNA repair proteins examined in the present work, are present in surface loops of proteins, which are generally involved in mediating novel protein-protein or protein-DNA (ligand) interactions [5,37,39,40]. While the possible cellular functions of most of the CSIs identified in this study remains to be delineated, a number of novel observations reported here provide important insights into the possible cellular function of a large CSI found in the UvrA1 protein. The UvrA1 protein is a central component of the NER pathway comprising of the UvrABC proteins [12,47,48,87]. This protein is unique in its ability to recognize a broad range of DNA damages including cyclobutane pyrimidine dimers and 6,4-photoproducts formed by UV light and multiple other types of damaged nucleotides/bases resulting from exposure to ionizing radiation [12,47,48,87]. Following, initial DNA damage identification by UvrA1, other proteins in the pathway viz. UvrB and UvrC, excise the damaged region and the gap created is filled by DNA polymerase I and subsequently ligated by DNA ligase [12,47,48,87]. Although *Deinococcus* species contain two different UvrA homologs, only the UvrA1 protein but not UvrA2, plays an important role in DNA repair process [80]. The gene for UvrA1 protein is also induced upon radiation and desiccation [12]. The present work has identified two novel characteristics of the UvrA1 protein that are uniquely observed for *Deinococcus* species. First, the UvrA1 protein from *Deinococcus* contains a 30 amino acid insertion in a conserved region that is absent in all other UvrA homologs (including UvrA2). Second, the gene for UvrA1 in all *Deinococcus* species is linked to the gene for a novel protein (dCSP-1) that is only found in different *Deinococcus* species. Additionally, in most *Deinococcus* species, except *D. radiodurans* and *D. wulumuqiensis*, the genes for *uvrA1* and *dCSP-1* are also specifically linked to the genes for DsbA and DsbB proteins and all four of these genes are predicted to form an operon. This novel arrangement/linkage of genes i.e., *uvrA1-dCSP-1-dsbA*-*dsbB* is only seen in *Deinococcus* species but in no other bacteria.

Of the three proteins genetically linked to UvrA1, dCSP-1 is a transmembrane protein, similar to the DsbB protein. The other two proteins, DsbA and DsbB, are both involved in the formation of intrachain disulfide bonds in proteins by catalyzing oxidation-reduction of cysteine residues in protein sequences. Of these, DsbA is localized in periplasm, whereas DsbB is an integral cytoplasmic membrane protein. As most of the proteins showing genetic-linkage to the *Deinococcus* UvrA1 are either cytoplasmic membrane or periplasmic proteins and two of them whose functions are known are involved in the oxidation-reduction of cysteine residues in proteins, it focuses attention on the significance of cysteine oxidation-reduction and membrane association in the functioning of UvrA1 protein. There is now considerable evidence that proteins are the major initial targets of free radicals or ROS in comparison to either DNA or lipids [19,29,30,89]. In proteins, cysteine residues, when present, generally serve important catalytic, regulatory, structure-stabilizing, or metal and cofactor binding functions and they are highly susceptible to modification by reactive oxygen species [53,91]. Many cytosolic proteins involved in catalyzing oxidation-reduction reactions contain CXXC motifs and the Cys residues in them exist as highly-reactive thiolate (S^−^) ions, whose oxidation can result in the functional inactivation of proteins [30,53,91,92]. In this context, it is of much interest that cysteine residues and CXXC motifs are present in a large number of DNA repair proteins (Table A1 and Figure S17). Of particular interest in this context is the fact that of all the DNA repair proteins, maximal numbers of CXXC motifs (5 in comparison to 0–2 found in other proteins) are present in the UvrA1 protein and they are parts of zinc finger elements, which play important roles in the binding of UvrA to DNA and in DNA damage recognition [54,55,56,88]. The above characteristics of the UvrA protein make it a prime target to be affected by oxidative stress and ROS. The importance of cysteine residues in the functioning of UvrA protein is also supported by a number of other observations: (i) Substitution of a cysteine in one of the C-terminal CXXC motifs causes inactivation of the UvrA protein [54]; (ii) Treatment of *D. radiodurans* with iodoacetamide (IAA), which alkylates –SH groups in cysteines, abolishes or greatly reduces its radiation resistance [93]; (iii) Treatment with IAA also causes repression (or inactivation) of a protein that excises DNA from a DNA-membrane complex [94,95]; (iv) Irradiation of *D. radiodurans* in presence of cysteine, which should protect Cys residues in proteins from oxidation, decreases their radiation sensitivity [96]; (v) Treatment of *Deinococcus* with sublethal concentration of cadmium leads to upregulations of a large number of genes involved in cysteine biosynthesis and disulfide stress indicating the importance of Cys-related systems in resistance to oxidative stress [97]. A number of observations also indicate that the cellular function of UvrA involves interaction with membrane. Based on earlier studies DNA in unirradiated *Deinococcus* is bound to membrane and it dissociates from membrane upon radiation treatment; importantly the re-association of DNA with membrane is inhibited by IAA [94,98,99]. Further, it has been reported that following UV irradiation, many DNA repair proteins relocate to the inner membrane and UvrA protein serves as a site of attachment for these proteins to the membrane [100].

**Figure 9:**
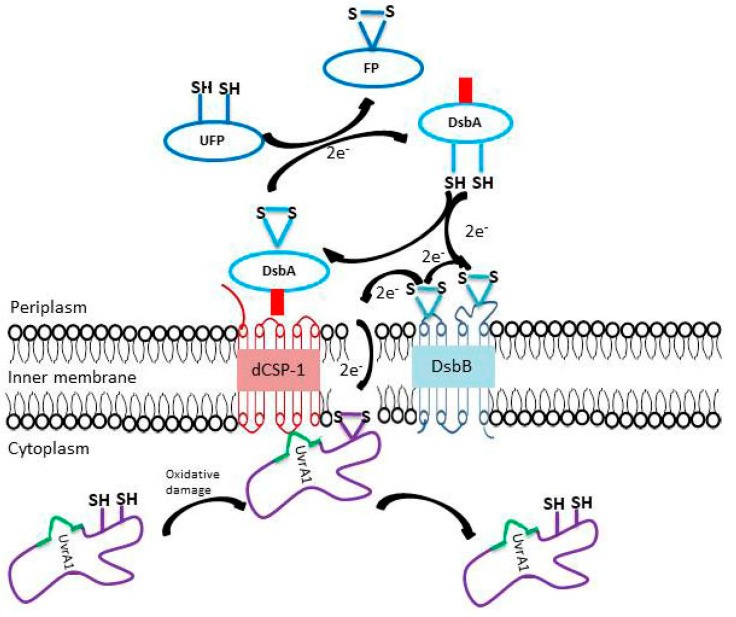
Proposed model to account for the novel genomic-proteomic characteristics of the UvrA1 gene/protein from *Deinococcus* species and their possible roles in protection of this protein from oxidatively generated damage. The model shown here proposes that the 5–7 amino acid insert present specifically in the *Deinococcus* DsbA protein (shown in red) plays a role in its interaction with the membrane embedded *Deinococcus*-specific protein dCSP-1. The model also suggests that the 30 amino acids insert found uniquely in *Deinococcus* UvrA1 homologs allows specific interaction with the dCSP-1 protein. These interactions position the UvrA1 protein in the proximity of cytoplasmic membrane such that the CXXC motifs in UvrA1 can receive electrons from DsbB protein to reduce oxidized cysteines and regenerate the functional non-oxidized form of the UvrA1 protein. The arrows indicate the direction of electron flow from DsbA to DsbB and to the membrane-associated UvrA1 protein. FP and UFP refer to folded and unfolded proteins.

Based on the above observations, to account for the different novel properties of the UvrA1 gene/protein from *Deinococcus* species reported here and how they may serve to protect this protein from oxidatively generated damage, we are proposing a model shown in Figure 9.

In this model, the proteins DsbA and DsbB, both of which contain CXXC motifs, are located in periplasm and cytoplasmic membrane, respectively, performing their well-studied functions in the oxidation-reduction of disulfide bonds in proteins [50,51,101,102]. A CXXC motif present at the active site of DsbA serves as the primary donor of disulfide bond to other unfolded proteins (UFP) in the periplasmic space. The reduced form of DsbA is reoxidized by transfer of electrons to the CXXC motifs in the DsbB protein and reducing it [50,51,101,102]. The DsbA protein in *Deinococcus* contains a 5–7 amino acid insert (shown in red) that is uniquely found in these bacteria. The model proposes that one possible function of this insert could be to enable specific interaction between the DsbA protein of *Deinococcus* and the membrane embedded dCSP-1 protein, which is also uniquely found in these bacteria. Another novel characteristic of *Deinococcus* identified in this work is the 30 amino acid insertion in the UvrA1 protein (shown in green in Figure 9). We are suggesting that one possible function of this insert in UvrA1 is to enable specific interaction with the dCSP-1 protein, thereby linking the UvrA1 to the dCSP-1, DsbA and DsbB proteins. The observed close genetic linkage of the *uvrA1* and *dCSP-1* also suggests the possibility that these two proteins are co-expressed under different conditions. It is suggested that the proposed interactions between the insert in the UvrA1 protein and dCSP-1 and the insert in DsbA protein and dCSP-1, the characteristics which are distinctive of *Deinococcus* species, serve to position the UvrA1 protein in *Deinococcus* in the proximity of membrane-bound DsbB protein (Figure 9). The reduced form of DsbB generally transfers electrons to the terminal oxidases via the quinone cofactor [50,51,101,102,103]. However, in *Deinococcus* species, we are proposing that due to the specific association of UvrA1 with the membrane embedded dCSP-1, electrons transfer can occur from DsbB (either directly or through quinone intermediate) to the oxidized CXXC motifs in the UvrA1 protein, thereby removing oxidative damage from this critical protein and restoring it into its non-oxidized functional state (Figure 9).

It should be noted that while DsbA homologs from most *Deinococcus* species contain a 7 amino acid insertion (Figure 7), the insert in *D. radiodurans* and *D. wulumuqiensis* is 5 amino acids long. In these two *Deinococcus* species, the genes for UvrA1 and dCSP-1 are also not genetically linked to the genes for DsbA and DsbB proteins but instead they exhibit a close genetic linkage to the gene for another novel protein dCSP-2, which is only found in these two *Deinococcus* species. The protein dCSP-2 is also predicted to be a membrane-associated protein and it is possible that this protein functioning in conjunction with the shorter CSI found in the DsbA homologs of these species, enables/augment specific interaction between the DsbA and dCSP-1 proteins in these two *Deinococcus* species.

In addition to the unique genetic linkage of the *Deinococcus* UvrA1 to the membrane associated dCSP-1, DsbA and DsbB proteins, a number of other important DNA repair proteins in *Deinococcus* species contain novel sequence features and some of them exhibit unique genetic linkages to membrane-associated proteins. We have shown in this work that the UvsE protein, central to the UvsE-dependent pathway of excision DNA repair [12,49], also contains a 1 amino acid CSI that is distinctive of *Deinococcus* homologs and its gene exhibits a novel operonic arrangement in *Deinococcus* with the gene for a Zn-ribbon (Zn-R) protein, which is predicted to be a transmembrane protein (Figure 5). Further, it has been reported that the gene for RecA in *D. radiodurans* forms a polycistronic operon with the genes for CinA and LigT proteins [104]. Our analysis indicates that a specific genetic linkage of the genes for RecA, CinA and LigT is a shared characteristic of all *Deinococcus-Themus* species (see Figure S16). Further, it has been reported that the CinA protein binds RecA and locates it to the cell membrane [105]. Thus, it is possible that the membrane association of UvsE and RecA proteins, seen specifically in *Deinococcus* species may also serve to protect these proteins from oxidatively generated damage.

In summation, the present work has identified many novel sequence features in the DNA repair genome/proteome of *Deinococcus* species which are predicted to contribute towards the increased resistance of these organisms to radiation/desiccation and other oxidative stress inducing agents. While the model proposed in Figure 9 is consistent with a large number of observations, it is primarily based on novel sequence and structural characteristics of the UvrA1 protein from *Deinococcus* and other proteins whose genes are genetically linked to the UvrA1 protein in these bacteria. It would be important to confirm various aspects of the suggested model by means of experimental approaches. However, it should be noted that one observation which conflicts with the present model concerns the report that the *uvrA* gene from *E. coli* (which is similar to the *uvrA2* gene found in *Deinococcus*) can complement the mitomycin C-sensitive phenotype of some *D. radiodurans* mutants [106]. This observation is surprising in view of the various novel sequence features of the UvrA1 gene/protein from *Deinococcus* species identified in this work, which distinguish it from all other homologs. Earlier work on CSIs and CSPs strongly indicates that these characteristics are functionally important for the group of organisms for which they are specific and deletion or mutational changes in them generally leads to functional inactivation [38,39,90,107]. Based on this, it is expected that the novel sequence features of the UvrA1 protein identified here should also serve important functions in *Deinococcus* species and that other UvrA homologs lacking these novel features, including the UvrA2 homolog from *Deinococcus*, should not be able to serve similar function. Thus, the ability of the *E. coli uvrA* gene to replace/complement the function of the *uvrA1* gene of *Deinococcus* is contrary to these expectations and it needs to be investigated more thoroughly. The possible cellular functions of CSIs in other DNA repair proteins, which are specific for *Deinococcus* species also remains to be determined and further studies on them could provide other useful insights into novel functional aspects of other DNA proteins in *Deinococcus*.

## Figures and Tables

**Table 1:** Summary of the *Deinococcus*-specific Conserved Signature Indels (CSIs) in DNA Repair Proteins.

Protein Name	Repair Pathway	Protein ID ^a^	Indel Size	Indel ^b^ Position	Figure
UvrA1	NER	DR1771	30 aa ins	421–514	Figure 1A
UvrC	NER	DR1354	16 aa ins	459–535	Figure 1B
UvrD	MP	DR1775	6 aa ins	516–576	Figure 2A
UvsE	UVER	DR1819	1 aa ins	48–125	Figure 2B
MutY	BER	DR2285	4 aa ins	24–82	Figure S1
MutM	BER	DR0493	2 aa del	168–225	Figure S2
Endonuclease III (Nth)	BER	DR0928	2 aa ins	144–228	Figure S3
RecA ^c^	HR	DR2340	1 aa ins	216–280	Figure S4
RecR ^c^	HR	DR0198	2 aa del	104–164	Figure S5
DNA helicase (RecQ)	HR	DR1289	2 aa del	453–510	Figure S6
Helicase RecD protein	HR	DR1902	2 aa del	376–427	Figure S7A
Helicase RecD protein	HR	DR1902	2 aa del	426–493	Figure S7B
RuvC protein	HR	DR0440	2 aa del	82–147	Figure S8
DNA helicase RecG protein	HR	DR1916	1 aa ins	14–73	Figure S9
DNA Repair protein RadA	HR	DR1105	1 aa del; ^c^	175–195	[5]
			2 aa del	225–257	
DNA polymerase I (PolA)	MP	DR1707	2 aa ins	191–257	Figure S10
DNA polymerase III, α subunit (DnaE)	MP	DR0507	65 aa ins	315–491	Figure S11
DNA polymerase III, α subunit (DnaE)	MP	DR0507	2 aa ins	75–131	Figure S12
DNA ligase (LigA)	MP	DR2069	3 aa ins	101–169	Figure S13
Gyrase A (GyrA)	MP	DR1913	1 aa ins	265–341	Figure S14
Gyrase B (GyrB)	MP	DR0906	1 aa ins	27–99	Figure S15

**^a^** Protein ID corresponds to the *Deinococcus radiodurans* genome; **^b^** The indel position indicates the region of the protein where the CSI is found. Ins and del indicate whether the CSI is an insertion or a deletion; **^c^** Indel is specific for Deinococcus-Thermus phylum; Abbreviations: BER- Base excision repair; NER-Nucleotide excision repair; HR- Homologous recombination; UVER-UV damage endonuclease (UvsE)-dependent excision repair; MR- Mismatch repair; MP-Multiple pathways. The CSIs in RadA were identified in earlier work [5].

## Supplementary Materials

The following are available online at www.mdpi.com/2073-4425/9/3/149/s1. Figure S1: Partial sequence alignment of MutY protein showing a 4 aa CSI specific for *Deinococcus*; Figure S2: Partial sequence alignment of MutM protein showing a *Deinococcus*-specific CSI; Figure S3: Partial sequence alignment of Endonuclease III (Nth) protein showing a showing a *Deinococcus*-specific CSI; Figure S4: Partial sequence alignment of RecA protein showing a showing CSI specific for Deinococcus-Thermus; Figure S5: Partial sequence alignment of RecR protein showing a showing a showing CSI specific for Deinococcus-Thermus; Figure S6: Partial sequence alignment of DNA helicase RecQ protein showing a *Deinococcus*-specific CSI; Figure S7: Partial sequence alignments of RecD protein showing two 2 CSIs that are specific for *Deinococcus*; Figure S8: Partial sequence alignment of RuvC protein showing a *Deinococcus*-specific CSI; Figure S9: Partial sequence alignment of RecG protein showing a CSI that is specific for *Deinococcus* species; Figure S10: Partial sequence alignment of DNA polymerase I (PolA) showing a CSI that is specific for *Deinococcus* species; Figure S11: Partial sequence alignment of DNA polymerase III alpha (DnaE) showing a CSI that is specific for *Deinococcus* species; Figure S12: Partial sequence alignment of conserved region of DnaE protein showing another CSI that is specific for *Deinococcus* species; Figure S13: Partial sequence alignment of LigA protein showing a CSI that is specific for *Deinococcus* species; Figure S14: Partial sequence alignment of DNA gyrase A (GyrA) protein showing a 1 amino acid CSI that is uniquely shared by all *Deinococcus* homologs; Figure S15: Partial sequence alignment of GyrB protein showing a CSI that is specific for *Deinococcus* species; Figure S16: Genomic neighborhood of the *recA* gene from representative Deinococcus-Thermus spp.; Figure S17: Sequence alignment showing the presence of CXXC motifs (highlighted) in different DNA Repair proteins.

## Appendix A

**Table A1:** Presence of Cysteine Residues and CXXC Motifs in DNA Repair Proteins.

Protein Name	Protein ID	# Of Cys Residues	CXXC Motifs	Figure Number
UvrA1	DR1771	11	5	Figure S17A
AlkA	DR2074	3	-	-
Endonuclease V (Nfi)	DR2162	1	-	-
MutM	DR0493	5	2	Figure S17B
8-Oxoguanine DNA glycosylase (MutY)	DR2285	6	1	Figure S17C
Endonuclease III (Nth)	DR0928	6	1	Figure S17D
Exonuclease III (Xth)	DR0354	2	-	
UvrB	DR2275	6	-	-
UvrC	DR1354	3	-	-
UvsE	DR1819	4	-	-
Rad25	DRA0131	10	2	Figure S17E
RecA	DR2340	1	-	-
RecO	DR0819	4	2	Figure S17F
RecR	DR0198	5	2	Figure S17G
RecJ	DR1126	2	-	-
RecN	DR1477	1	-	-
RecQ	DR1289	9	1	Figure S17H
RecD	DR1902	1	-	-
SbcC	DR1922	4	1	Figure S17I
SbcD	DR1921	1	-	-
RuvA	DR1274	1	-	-
RuvC	DR0440	2	-	-
RecG	DR1916	4	-	-
RadA	DR1105	5	2	Figure S17J
Rad54 DNA helicase	DR1259	3	-	-
DdrA	DR0423	2	-	-
MutL	DR1696	4	-	-
MutS	DR1039	4	-	-
PolA	DR1707	5	-	-
DNA polymerase III, α subunit (DnaE)	DR0507	14	-	-
DNA polymerase III ε subunit (DnaQ)	DR0856	1	-	-
DNA polymerase III subunit beta	DR0001	1	-	-
DNA ligase (LigA)	DR2069	4	1	Figure S17K
DNA polymerase III subunit gamma/tau	DR2410	7	1	Figure S17L
UvrD	DR1775	1	-	-
Gyrase A(GyrA)	DR1913	1	-	-
Gyrase B(GyrB)	DR0906	2	-	-

All BLASTp hits showing significant similarity to the dCSP-1 protein (accession number NP_295493) are for *Deinococcus* species.
