# Supplementary material for: Novel Sequence Features of DNA Repair Genes/Proteins from Deinococcus Species Implicated in Protection from Oxidatively Generated Damage
Source: Genes (Basel). 2018 Mar 8;9(3):149. doi: 10.3390/genes9030149 (PMC5867870; doi:10.3390/genes9030149)
Supplement: Supplementary file 1 [file genes-09-00149-s001.pdf]

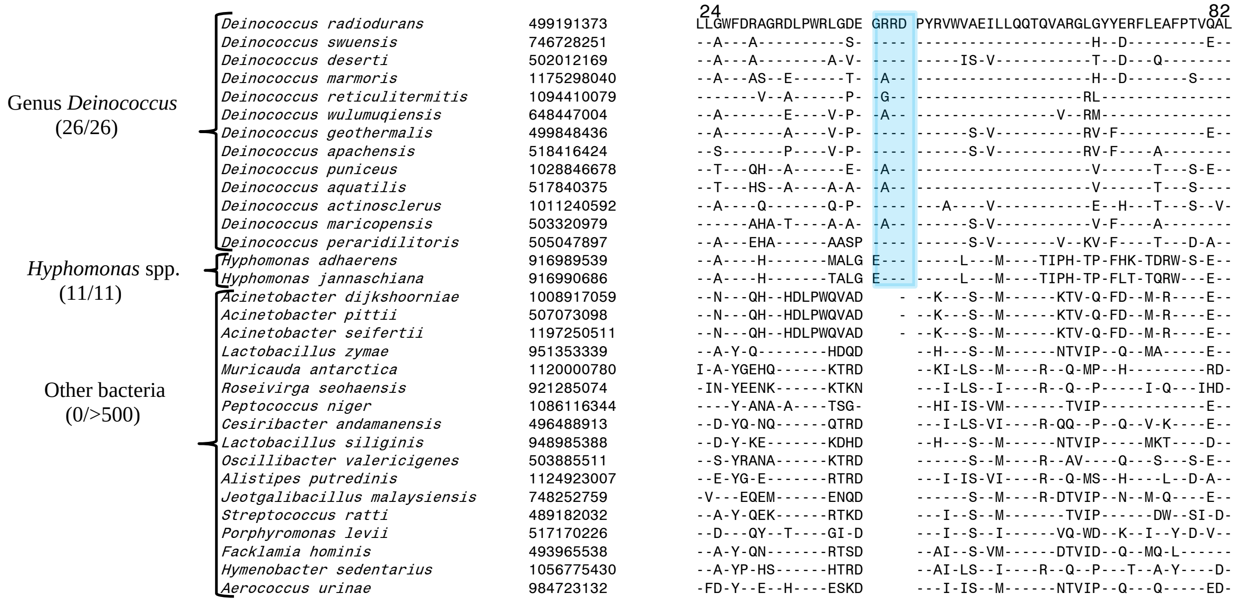

Figure S1. Partial sequence alignment of conserved region of 8-oxoguanine DNA glycosylase (MutY) protein showing a 4 amino acid insertion that is a distinctive characteristic from homologs of *Deinococcus* group of bacteria. This insert is also shared by *Hyphomonas* spp. and it has likely occurred independent in this group.

Group	Species	ID	168 … 225
Genus *Deinococcus* (26/26)	*Deinococcus radiodurans*	24211712	KPWLLSQKPVSGVGNIYADESLWHARLHPAQTRLNADEAG RLYRAIREVMAAAVDKGG
	Deinococcus gobiensis	504497204	--------------------A-----I-----A------S -----V-----Q--EA--
	Deinococcus deserti	502015685	----------------------M--I-------K----R ---Q------T---EA--
	Deinococcus aquatilis	517838810	----------------------T-QI--------P---T ---H------G---EA--
	Deinococcus geothermalis	499848854	----------------------R--I-----N-S-P--- -----------E--AA--
	Deinococcus puniceus	1028846612	----------------------T-QI---------A--T ---H------G---EA--
	Deinococcus hopiensis	1180560487	--------------------A--R----------TR---- ---Q-V---LGE--EA--
	Deinococcus apachensis	518417311	----------------------R--I-------TRE--- ---H-V----HE--EA--
	Deinococcus reticulitermitis	1094409411	----------------------L----------TEG--A ---H-L-G---E--ER--
	Deinococcus actinosclerus	1011238849	----------------------L-G---T----TR---- ---H-V-D--GR--EA--
	Deinococcus marmoris	1132147771	----------------------Q-QI-------TSA-GK K--T-------K--EA--
	Deinococcus wulumuqiensis	516482020	-V--------------------LSKI-----K-T----S ---H-V----HE--ER--
	Deinococcus maricopensis	503322817	-------V--A-----------R--I----R--TL---T --HA-------E--QA--
	Deinococcus pimensis	653295286	-------V--A-----------R--I-------TRE--V --RA-------E--AV--
	Deinococcus proteolyticus	503380136	-------R--A---------A--R--I----RH-S-EQ-A --HA-V----RE--EL--
Other Deinococcus-Thermus (0/27)	*Truepera radiovictrix*	754553609	--F-----------------A--R--I--LTPANEVSR-K AA Q-AG-----L--SLRAK-
	Oceanithermus profundus	503223321	--A----E--A-L-------A--K------RPANALAPDE VR --H--V-A-LRR--ER--
	Meiothermus rufus	654411381	-EV--G-EA-A-----------MSKI--ERPAGSLRPNE VR ---Q-------R--EA--
	Meiothermus timidus	517278211	-EV--G-EA-A-----------MS-I--ERPACSLRLEE VE ---Q------GR--SA--
	Meiothermus silvanus	502922961	-EV--G-EA-A-I----------QS-I--ERPANTLKP-E VK ---K---D--GR--EA--
	Meiothermus ruber	738244021	-EV--A-EA-A-----------LSQI--ERPAASLSSPE VR ---K---V--ER--EA--
	Marinithermus hydrothermalis	503468993	-AV--A-EV-A-L-------A--R-GV---RPANTLEVGA IA ---T-----L-E--AA--
	Thermus thermophilus	499487190	-AL--D-RLAA---------A-FR---S-FRPARSLT-EE AR -----L---L-E--EL--
Other bacteria (0/>500)	*Enterococcus faecalis*	504675046	--L--D--L-T-L----V--A--Q-QI--E-PADSLKP-E VA K--Q--ID-L-R--EA--
	Enterococcus ureilyticus	1069232050	--L--D-RL-T-L----V--A--E-KI--E-PADTLKAKE IE ---H--ID-LGR--EA--
	Chlamydia trachomatis	815078700	--TI-D-SVIA-----------E-KI--ETRVNKLSSVD LE N--K---K--KISIQR--
	Nitrolancea hollandica	916271686	--V--D-SV-A-L----V-----------ERVAGSLS-EE IE ---TG-NQII----PM--
	Tetragenococcus solitarius	1057346088	--L--N--V-A-L----T--V--L-KI--Q-PAQTLNKKE SK ---Q--ID-L-K--RA--
	Thermorudis peleae	917306931	--V--D-RV-A-L----V-----Q-Q----RRVGTLTLPE ID ---E--KT--EV--PI--
	Enterococcus rivorum	1070078037	--L--D--L-T-L----V--A--E-KI--E-PANTLKPKE VE E--Y--ID-L-R--EA--
	Melissococcus plutonius	503540406	--L--E--L-T-L----V--A--T-KI--EKPANTLSVSE VN --HK--IDILTQ--KA--
	Tetragenococcus muriaticus	675813703	--L--N--V-V-L----T--A--T-KI--Q-PASTLTHRQ IT K-HQS-I-IL-K--NA--
	Plantibacter flavus	1188389498	-RA--D-TL---I----------A--I--E-ETARLGLGR AG --LEEV-A-L-K-LAE--
	Enterococcus ureasiticus	1069255546	--L--D-RL-T-L----V--A--E-KI--E-PADTLKPKE VE Q--Y--ID-LGC--EA--
	Tetragenococcus halophilus	1007070729	--L--N--V-A-L-------V--L-KI--Q-PARTLTKKE AE S-HQ--IAIL-K--RA--

Figure S2. Partial sequence alignment of conserved region of formamidopyrimidine and 8-oxoguanine DNA glycosylase (MutM) protein showing a 2 amino acid deletion that is a distinctive molecular characteristics of *Deinococcus* spp.

			144		228
Genus *Deinococcus* (26/26)	*Deinococcus phoenicis*	736331005	GVGMKTASLLLLFDLARPAIPVENNIHRVAGRLDLIPARWNVLKAERWFDGVLPRD	WA	ARATFHVSAIRHGRQTCLSQRPRCEVC
	Deinococcus geothermalis	499849194	---------------------I-------------F-S------------E-----	-L	D----------------RA-----AC-
	Deinococcus apachensis	518415322	--------------------------W-----------------------E-----	--	T-Y----------------R-------
	Deinococcus puniceus	1028846869	---------V---------M--DTH-G-I-A--EWV-----AI-V-----E-----	--	S-YG----------E--RA---D--A-
	Deinococcus marmoris	736389575	--------CV---------M--DTH---I-R--E-V-EA--AV-V-----E----E	--	--Y--------------RARN-A-G--
	Deinococcus maricopensis	503322632	--------A----------M--DGH-D--SK--H---E----------Y-E-----	--	Q-YAY--AT-----E---TRA---NA-
	Deinococcus deserti	613465786	---------I-----L---L--DT--E-I-K--E-V-Q--TPE-V-----A-VR--	--	E------AGV----L--RPRD---DQ-
	Deinococcus wulumuqiensis	516480559	---H--VA-V-----R---M--DG-ME-A-K--E-V--A--SH-V---YAE-V-G-	-E	T-FAL-I-GV----D--R-KH-L-PA-
	Deinococcus ficus	760096023	---L-----V-----I---L--DG--E-TLK--EFV-PN-SAERT-----R-VS-E	--	L--AL--AGV----H---PRN---PA-
	Deinococcus gobiensis	504498115	---QR----V-----V---M--DT--A-M-A----V-ET-STNRT-A--GQ-IA--	-E	T-YAL-L-GV---HE--TPR--L-GR-
	Deinococcus radiodurans	499190033	---H--VA-V-----R---M--DG-ME-A-K--E-V--A--SH-V---YAE-M-A-	-E	T-FAL-I-GV----D--R-KH-L-PQ-
	Deinococcus reticulitermitis	1094409371	---H--VA-V-----G-A-M--DG-ME-A-K--E-V--G-SSDRV---Y-EAA---	-E	T-FAL-L-GV----V--RP---L-GE-
	Deinococcus proteolyticus	503380505	---QR--------H--Q--AA-DS--E-LLH--EVV-PG-KADRQ-L-LE----A-	AP	L--A--RAGV----EI-TRHA---PA-
Other bacteria (0/>500)	*Thermorudis peleae*	1175339156	---P---ACV---G-G---L--DTHVY---Q--G-L-P-CTSER-HQLLAALV-PE		Y--A---LL------C-HARN---PT-
	Methanosaeta harundinacea	504400110	---P---AVV---AFRM-LL--DTHVN-LSR--GFV--GASIEE---ILEEIT--E		KYCS---NL-----AV-RARS-S-GA-
	Ktedonobacter racemifer	495198690	---P---ACV---NMG--LM-IDTHL--LTH--G--GPKVSADQ-HTI-LKA--PE		WAY-L--NL-----TI-HA---K-PQ-
	Anaerolinea thermolimosa	1011275067	---P---AIV-V-S-NK--F--DTH-Y--S--IG-R--HLS-EQ-HQYLA--FKP-		QY-PG-LNL--L--E--HAR--N-PA-
	Chloroflexi bacterium	1084570780	---P---AIV---S-N---F--DTH-Y--T--IG-R--KMT-EQ-HPYLESLF-A-		-YYAA-LNI--L--EV-QAR-TM-YK-
	Dehalococcoidia bacterium	931359859	---P---ACV---S-G-SVL--DTHVY-ISR--G--DS-VSPEQ-HQLLEE-V-SQ		-LYQ--LNMLA---SI-RA---L-HD-
	Chthonomonas calidirosea	944158928	---P---AIV-C-A-G--V---DTHVF---W--G--EK-VGES--HDLLQALV-PE		LIYR---AL-E---RV-KAL-------
	Thermogemmatispora onikobensis	1181357612	---P---ACV---A-GW-VM--DTHV----R--G-LGPKVSAEQ-HVLLAQMT-PA		WVYAL--NL-----RV--A-----PA-
	Anaerolineae bacterium	931423785	---R----IV---SFG---F--DTHV--IS---G--GPKVTAD--HQILENMGDP-		TYYAM-LNL-----EV-TARN-K-DQ-
	Solirubrobacterales bacterium	1113216706	---R---ACV-I-SWGL-E---DVH----G---G-F--KASLER-HDEMLAIV-PE		DAYEL--NL-----TL-RP K---GG-
	Euryarchaeota archaeon	1197629305	-I-P-SSAVI-N--FDKN-F--DTHVY--TQ--G---NKT-RE--HQILEKQV-SE		RMYE--INL-K---TV-KARK-I-SE-
	Anaerolineaceae bacterium	1176212619	---P---AIV---SFNL--F--DTHV---SQ--G--GTQVSRE--HSTLETL--PE		TYYS--LNL-A----V-HARG----H-
	Anaerolinea thermophila	503323788	---V----IV---S-NK--F--DTHV---S--IG-R-PQMSAED-HAYLAQ-FTPE		QY-AG-LNL--L--EV-HARK-A-PR-

Figure S3. Partial sequence alignment of conserved region of Endonuclease III (Nth) protein showing a 2 amino acid insertion that is specific for all *Deinococcus* group of bacteria.

Group	Organism	ID	216		280
Phylum Deinococcus-Thermus (53/53)	*Deinococcus radiodurans*	499191426	GNPETTTGGRALKFYASVRLDVRKIGQ	P	TKVGNDAVANTVKIKTVKNKVAAPFKEVELALVYGKG
	Deinococcus wulumuqiensis	516482149	---------------------------	-	-------------------------------------
	Deinococcus maricopensis	503323319	---------------S-----------	-	-------------V-----------------------
	Deinococcus gobiensis	504499138	---------------------------	-	I------------V-----------------------
	Deinococcus puniceus	1028844635	---------------S-----------	-	I------------------------------------
	Deinococcus aquatilis	517841573	---------------S-----------	-	I------------------------------------
	Deinococcus marmoris	657681647	---------------ST----------	-	I--------------------------------F---
	Deinococcus frigens	657673652	---------------ST----------	-	I--------------------------------F---
	Deinococcus deserti	502016032	---------------------------	-	V-L-----G----V-S------P-------T-M----
	Deinococcus peraridilitoris	505049365	---------------------------	-	M-S-----G------V------P--Q------IF---
	Deinococcus geothermalis	499850523	---------------------------	-	V-L-----G----V--------P-------T-L----
	Meiothermus timidus	648542800	------P----------------RQ--	-	I-------G-R-RV-VT-----P--R-A-IE-Y----
	Meiothermus chliarophilus	654422710	------P----------------RQ--	-	I-------G-R-RV-VT-----P--R-A-IE-Y----
	Meiothermus silvanus	502922380	------P-----------------Q--	-	I------IG-R--V-VT---L-P--R-H-IE-YF---
	Meiothermus ruber	738243925	------P-----------------Q--	-	I-------G-R-RV-VT---L-P--R-H-IE-YF---
	Meiothermus cerbereus	654400463	------P-----------------Q--	-	I-S-----G-R-RV-VT---L-P--R-H-IE-YF---
	Meiothermus rufus	654410269	------P-----------------Q--	-	I-S--E--G-R-RV-VT---L-P--R-H--E-YF---
	Marinithermus hydrothermalis	918004604	------P----------------RS--	-	I-Q--E-IG-R-RV-VT---L-P--R-A-IE-YF---
	Oceanithermus profundus	503222090	----V----------S-I-----RK--	-	I-K-DQPI--V-RV-VT---L-P--R-A--E-YF-R-
Other bacteria (0/>500)	*Escherichia coli*	338843449	---------N-----------I-R--A		V-E-ENV-GSETRV-V----I-----QA-FQIL--E-
	Shewanella benthica	161330260	---------N-----------I-R--A		I-N-DEVIG-ETRV-V----I-----QA-FQIL----
	Acetonema longum	490710638	--------------------E--RTES		I-------G-RT-V-V----I-P---QA-FDIM--E-
	Vibrio variabilis	324455904	---------N-----------I-RT-S		I-E-DEV-G-ETR--V----I-----QA-TQIL--Q-
	Salmonella enterica	544851066	---------N-----------I-R--A		V-E-DNV-GSETRV-V----I-----QA-FQIL--E-
	Salinivibrio siamensis	1145451095	---------N-----------I-R--A		I-E-DEV-G-ETRV-V----------QA-FQIL--Q-
	Tenericutes bacterium	1088626595	------P--------------I-RGE-		I-E---IIG-QARV-V----------VA-ID-IF---
	Paenibacillus rhizosphaerae	1133787499	------P--------S-------R-ES		I-M---V-G-RTR--V------P---QA-IDIM--E-
	Thermoanaerobacter mathranii	90568908	------P---------TI------VDP		I-Q--EI-G-RTRV-V------P---QA-FDIM--E-
	Nocardia farcinica	916346749	-S-------------------I-R-ET		L-D-T---G-RTRV-V------P---QA-FDIL--Q-
	Bifidobacterium longum	347369123	---------K-----------I-R-QT		L-N-DE--G-RTRV-V----M-P---SA-FDML--E-

Figure S4. Partial sequence alignment of conserved region of DNA recombination protein RecA showing a 1 amino acid insertion that is specific for all bacteria of Deinococcus-Thermus phylum.

Group	Species	Accession	104		164
Phylum Deionococcus-Thermus (53/53)	*Deinococcus radiodurans*	499189304	YHVLHGVLSPMNGVGPDKLHIKPLLPRVGQG		MEVILATGTTVEGDATALYLQRLLEPLGAAISRIAYGVPVGG
	Deinococcus reticulitermitis	1094407926	----------------E----R-------E-		-----------------------------DV------L----
	Deinococcus puniceus	1028846200	---------------------R------TE-		-----------------------------LV------L----
	Deinococcus gobiensis	504498386	----------------E----R------RE-		------------------------------V------L----
	Deinococcus aquatilis	517839910	---------------------R------QS-		-----------------------------VV------L----
	Deinococcus grandis	960504608	---------------------R------QE-		Q----------------------------VV------L----
	Deinococcus deserti	502013281	----------------E-----------QE-		Q-I--------------------------VV------L----
	Deinococcus murrayi	653254381	----------------ER---R------RE-		-----------------------------VV------L----
	Deinococcus swuensis	746724282	----------------EQ----------TE-		Q----------------------------TV------L----
	Deinococcus frigens	657673248	----------------EQ----------TD-		Q----------------------------LV------L----
	Deinococcus geothermalis	499849908	------A----------R-Q-R------QD-		-----------------------------VV------L----
	Deinococcus maricopensis	503321334	------AI-----------M--------AA-		-------S-----E--S------------TV------L----
	Deinococcus proteolyticus	503380241	----------------EQ----E----ARE-		L----------------------------EV------LQ--S
	Truepera radiovictrix	502943872	------A----------Q-T-Q---A-L-GV		R------A-----E---M--A---GE-KVPT------L----
	Thermus arciformis	1224989969	----G-A-N-LE----KE-NLES-W--LS-V		R--V---SM----E------AEE-KRR-VKA--L---L----
	Thermus caliditerrae	740205913	----G-A-N-LE----KE-NLET-W--LAGV		K--V---SM----E------AEE-KR--VKAT-L---L----
	Thermus aquaticus	489134392	----G-A-N-LE----KD-NLES-W--LQEV		R------SM----E----F-AEE-KKR-VKAT-L---L----
	Meiothermus cerbereus	654401139	----G-A-N-LE-I--EQ-NLEK-FS-LEGI		G-------M----E---GF-AER-NQMHIRST-L---L----
	Meiothermus rufus	654410713	----G-A-N-LE-I--EE-NLEG-WA-LEGV		Q------SM----E---S--AEC-RQRQIRST-L---L----
Other bacteria (0/>500)	*Acetanaerobacterium elongatum*	1086273160	------LI---D-I---EIY--E--E--K--	GI	K-I-V--NAN---E---M-IS---K---VKV--L---I----
	Ammonifex degensii	506220306	----------LD-------R-QE--Q-LQE-	EV	R------SS-T--E------K---K--EVKV--L---L---A
	Anaerotruncus rubiinfantis	1054688845	------LI---D-----Q-Y--E--S-M-S-	EI	A--VM--NP----E---M-IA---K-M-VKVT-L---I----
	Firmicutes bacterium	523988753	------------H----D-Q----VD--A--	GI	R---M--NPDT--ET-----A---K-F-VKVT-L---I----
	Intestinimonas butyriciproducens	959158736	-------I----H----D----E--E--AS-	EV	Q---M--NPDT--E---M--A---R-F-VR-T-L--------
	Clostridium leptum	524460415	-------I--LG-I---Q-K--E--D--NR-	GV	K---M--NP----E---M--S---K---IKVT-L---I----
	Anaerotruncus colihominis	493931685	------LI---D-I--EQ----E--A-ISA-	GV	D---M--NP----E---M-IA---K-M-VRVT-L---I----
	Anaeromassilibacillus senegalensis	914737187	------AI--L-----EQ-C--E--A-I-NS	EV	Q---M--NP----E---M-IS---K---VKVT-L---I----
	Intestinimonas massiliensis	914625887	-------I----H----D----E-ME--SG-	EV	R---M--NPDT--E---M--S---K-F-VRVT-L--------
	Angelakisella massiliensis	1100887849	------LI---E----EQ-T--E--A--NK-	GV	E---M--NP----E---M-IA---K---VKT--L---I----
	Ruminococcus flavefaciens	671586627	------L----D-IT----RV-E--A-IAK-	GV	E---M--NP----------VAG--K---VKV--L-F-L----
	Ruminococcus albus	1124684610	------LI--I---S--D-K--E--A-INS-	IA	D---M--NP--------F-ISK--K---VKVT-L-F-L-I--

Figure S5. Partial sequence alignment of conserved region of DNA helicase recombination protein RecR showing a 2 amino acid deletion that is a distinctive molecular characteristic of the phylum Deinococcus-Thermus.

Group	Species	ID	453		510
Genus *Deinococcus* (26/26)	*Deinococcus radiodurans*	499190392	HHQLPTFGVGKEHDEKLWRSVLRQLVSLGYLSAD		DHFGLRATGKSRGILKEGQKLLLR
	Deinococcus wulumuqiensis	516481428	--L-------R-----------------------		------------A-----------
	Deinococcus hopiensis	1180559944	----------SA----T--G--------A-----		E-Q-------A-AL--GEET-T--
	Deinococcus reticulitermitis	1094410232	--L-------RA-------------------AVG		-YS--G----A-AL-SG-E-----
	Deinococcus swuensis	746724908	--L--------G----M--GLI-------H---G		E-H--S------AL--GETT-M--
	Deinococcus deserti	502015129	----------R----RF--G-----T-----TSG		EYH--S----A-A---GEA--A--
	Deinococcus grandis	972317926	--T-------AA----T--G---------F-A-G		E-H--S----A-TL--GEAT-T--
	Deinococcus proteolyticus	503380202	--T-------A----RT--G----M------E-G		E-Y--S--P---EL--GEVPF---
	Deinococcus phoenicis	736325097	----------QG----A--GL----------A-G		E-H--S----A-PL--GEAT-H--
	Deinococcus murrayi	653257696	--T--------A----M--GL----------A-G		E-H--S--A-A-PL-RGEET-H--
	Deinococcus gobiensis	504499404	-R----Y---QG--V-V--------------T-G		PYQ--TV--RA----RGEVR----
	Deinococcus aquatilis	517838325	-RS---Y-I-----P-V--------------T-G		PYH--TV-PQAKA--RGETP----
	Deinococcus ficus	653264797	-RT-----I--D--ARV--------------T-G		PYQ--MV-A-AKYV--GERP----
	Deinococcus misasensis	736318342	-RT---Y-I-----A-V-G--I---------T-G		PFQ--MT-PQAKML-TGA-R----
Other bacteria (0/>500)	*Rhodanobacter fulvus*	494143086	----S-----ADM---Q----F---LAA-L-E--	AE	GYGT--L-AA---V-SG-ESVK--
	Thiothrix nivea	386421742	-D--S-Y-I-T--SQAE--NIF---IA-----V-	VD	G-G---L-D---PL-RGEIE-H--
	Bradyrhizobium erythrophlei	1089508368	-D--SV--I-R-LN--Q--A------AM-H-R--	SD	AFGA-KL-DSA--V--GETAVM--
	Thiothrix caldifontis	1088971155	-E--S---I----SDAE--NIF---IA----TV-	VD	G-G---L-DNA-PL-RGEIE-H--
	Dyella thiooxydans	1027736903	-DK-T-----ADM---A----F---LAA-L-AT-	PE	GYGT--L-AA---V-TG-ERV---
	Dyella ginsengisoli	648415568	-DK-T-----ADM---A----F---LAA-L-AT-	PE	GYGT--L-AA---V-TG-ERV---
	Haliea salexigens	654479105	--T-----I--AL-NHQ----F----AR---R--	LD	HFGA--LEDHC-PL-RGEET-E--
	Bradyrhizobium elkanii	740592818	-D--SV--I-R-LN--Q--A------AM-H-R--	SD	AFGA-VL-DSA--V--GETAVM--
	Bradyrhizobium pachyrhizi	913367243	-D--SV--I-R-LN--Q--A------AM-H-R--	SD	AFGA-VL-DSA--V--GETAVM--
	Thiothrix lacustris	740303120	-E--S-Y-I-T--SPEE--NIF---IA-----V-	VD	G-G---L-DN--PL-RGEIE-H--
	Nitrosospira briensis	640613188	-DKVS---I---L---A--A-F---AA--L-T--	SE	G-GS--LAAA--AV-TGN-TVR--

Figure S6. Partial sequence alignment of conserved region of DNA helicase RecQ protein showing a 2 amino acid deletion that is uniquely shared by all *Deinococcus* group of bacteria.

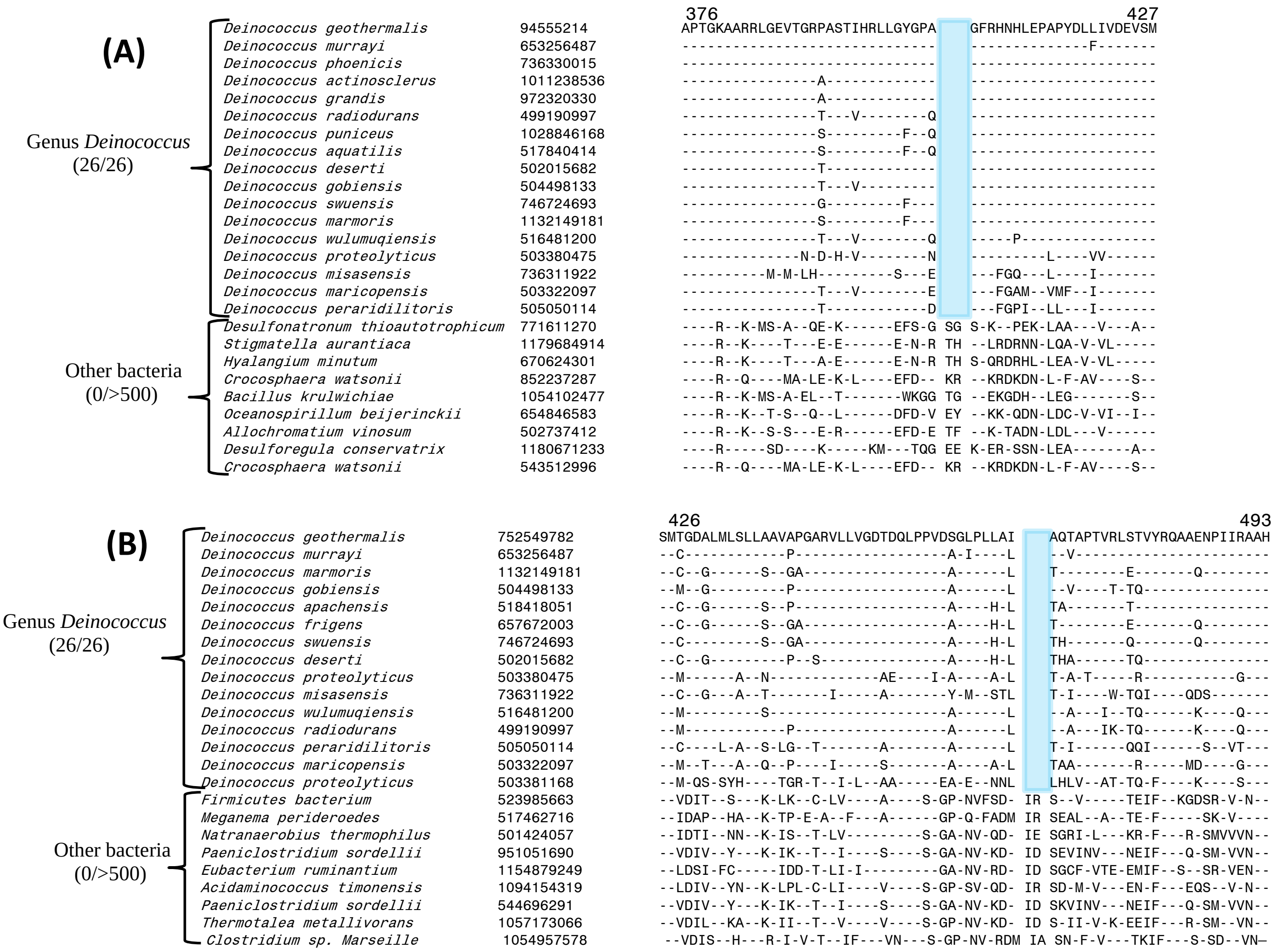

Figure S7. Partial sequence alignments of two conserved regions of helicase RecD protein showing two 2 amino acid deletions that are specific for all *Deinococcus* group of bacteria.

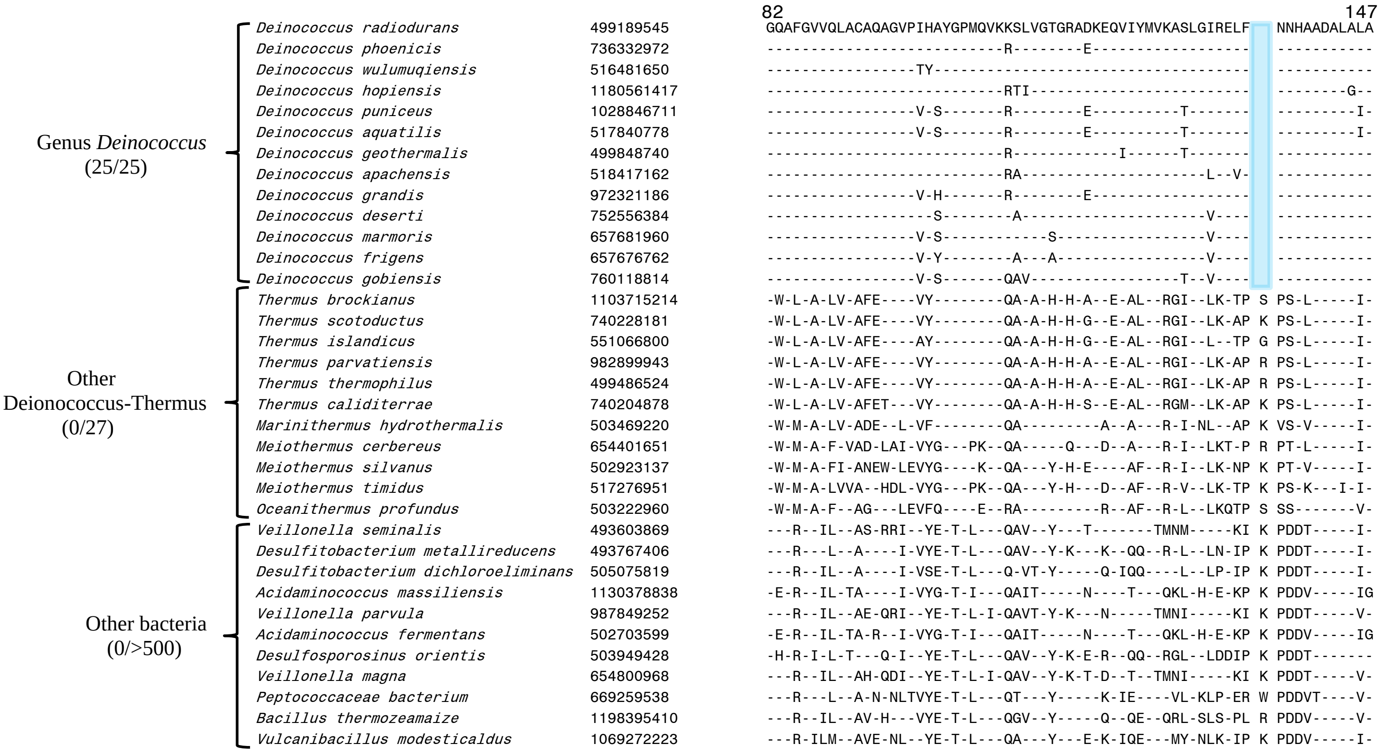

Figure S8. Partial sequence alignment of conserved region of crossover junction endodeoxyribonuclease RuvC protein showing a 2 amino acid deletion that is a distinctive molecular characteristic of *Deinococcus* spp.

			14		73
Genus *Deinococcus* (23/26)	*Deinococcus geothermalis*	499849539	PLAAELAAGCADRVVAGGVERLLAS	P	LAGPFPQVREALRGYAELDAAGRAAALHRALALL
	Deinococcus murrayi	653256037	-------S-----------------	-	------G---------G---GA--E--G------
	Deinococcus hopiensis	1180559598	----------G--------------	-	------G---V--------GVA--E--G---E--
	Deinococcus ficus	653259472	----------T---------K----	-	-GN---K-----A---G--P-A-E---TQ--E--
	Deinococcus radiodurans	499191011	-------G--H---------K--ST	-	------K---V-S---G--ESA--V--RE--T--
	Deinococcus actinosclerus	1011239130	-------S--QN--------K----	-	-GN---K-----A---A------EV--RA--D--
	Deinococcus grandis	972319627	-------S--QN--------K----	-	-GN---K-----A---G------EV--RA--D--
	Deinococcus wulumuqiensis	516481212	-------G--H---------K--ST	-	------R---V-S--GQMREDE--G--RE-----
	Deinococcus swuensis	746727406	-------G--QN-------DK----	-	-GN---KI--T----GD-GVTE-EE--KT---A-
	Deinococcus marmoris	657681888	----------QN-------DK----	-	-GN---KI--V-G--G--SVGE-EEV-KT---A-
	Deinococcus frigens	657673578	----------QN-------DK----	-	-GN---R---V-G--GA-SVSE-EDI-KT---M-
	Deinococcus deserti	502014968	----------QN--------K----	-	-GN-------V-G--GS-EP-K-E---KS-----
	Deinococcus marmoris	1131054793	----------QNH------DK----	-	-GN---KI--V-G--G--SVGE-EEV-KT---A-
	Deinococcus misasensis	736303153	--ER--LM--QNK-----L-K--DN		-GK---K---V--H-ET-PVEE-ETK-RQ--Q--
	Deinococcus pimensis	653295271	--ER--LT--TN--------K--DN		-GK-------T----S--EP----EK-RS-----
	Deinococcus peraridilitoris	505047055	--ER--LT--SN--------K--EH		-GK------------AM-EDE-GER-RA-----
Other Deionococcus-Thermus (0/27)	*Thermus thermophilus*	499547772	--LR--QD-AR----V--L-A-VQN		--R---KLL-LF---G-KPQEE-KRV-QE--R--
	Thermus arciformis	1086163620	--LR--QD-AR----V--L-A-VQN		--R---KLL-LF---G-RPPEE-KRV-QE--R--
	Thermus aquaticus	927054814	-ILR--QD-AR----V--L-G-VRN		--R---KLLDLFQ--G-K-PEA-KKV-EE--RI-
	Thermus islandicus	655041914	-ILR--QD-AR----V--L-G-VQN		--R---KLL-LF---G-RPPEE-KRI-EE--R--
	Oceanithermus profundus	503222827	--ER---T--R-------L-K-VHN		--R---E-I-L-E---A-EPDA-C-R-RE---R-
	Meiothermus cerbereus	916922623	-I-R---D-AQ-------L-K-IQN		-GQ---E-GRV-A--RQM-VEA-K-Q-KK--E--
	Meiothermus rufus	654411370	--QR---D-AR-------L----QN		-GH---ELGQV-A--RQM-P-T-R-R-EE--R--
	Meiothermus ruber	502779467	-I-R---D-AQ-------L-K-IQN		-GQ---E-GRL-A--RQM-VET-KEQ-KK--E--
	Meiothermus taiwanensis	738241890	-I-R---D-AQ-------L-K-IQN		-GQ---E-GRL-A--RQMEVEA-KER-IK--E--

Figure S9. Partial sequence alignment of a conserved region of DNA helicase RecG protein showing a 1 amino acid insertion that is specific for the *Deinococcus* group of bacteria. Three *Deinococcus spp.* do not contain this insertion.

Group	Species	Accession	191	Insert	257
Genus *Deinococcus* (26/26)	*Deinococcus radiodurans*	290068	DNIPGAKGIGPKTAAKLLQEYGTLEKVYEAA	HA	GTLKPDGTRKKLLDSEENVKFSHDLSCMVTDLPL
	Deinococcus wulumuqiensis	516480923	--------------------------I----	--	----------------QD-Q---S----------
	Deinococcus deserti	502015562	-------------------------GI----	R-	---------Q-------A-----E----------
	Deinococcus ficus	760095321	-------------------------GI----	K-	-------V-Q-------A-Q---E----------
	Deinococcus actinosclerus	1011239262	-------------------------GI-A--	K-	---------Q-------A-E---R----------
	Deinococcus grandis	972319813	-------------------------GI-A--	K-	---------Q-------A-E---R----------
	Deinococcus marmoris	1132147896	--------------S----D------IF---	--	---E-K---E---A-----Q---QM---------
	Deinococcus gobiensis	380000174	-------------------------GI-A--	--	-------A-Q---A--SD-Q---E----------
	Deinococcus geothermalis	499850060	---------------R--------DA-LA--	R-	---E-K---E---A--AD-L--RE----------
	Deinococcus hopiensis	1180560144	-------------------T-R--DA-LA--	KD	---E-K---E---T--AD-R---E------N---
	Deinococcus apachensis	518414954	-------------------Q----DT-L---	RS	---E-K---E---A--AD-L--R---R-------
	Deinococcus maricopensis	503321933	--------------------H-S-DA-LQ--	E-	-A---K--QE-IAA-VQD-L--RE----------
	Deinococcus murrayi	653254158	----------------I-------DAAL---	R-	---E-K---E---A--KD-LL-RE--R-------
	Deinococcus proteolyticus	503380145	--------------T-I-------DAALD--	K-	---Q-K---Q------DA-R--RE--Q--C--E-
Other Deionococcus-Thermus (0/27)	*Truepera radiovictrix*	502943864	-------------------D--S-DAIL-NL		ERV--ESAA--VRA-L-D----RE--RII--AD-
	Thermus antranikianii	655044621	-----V----E------IR-W-S--NLLKHL		EQV--ASV-E-I-SHM-DL-L-LE--RVY-----
	Thermus scotoductus	505921010	-----V----E------IR-W-S--NLLKHL		EQV--ASV-E-I-SHM-DL-L-LE--RVH-----
	Oceanithermus profundus	503223021	-----VR-V-A-------ARW-S-DRI--HL		DEVT-P-V----EEGR-AAF--REI-R-RA-V--
	Meiothermus silvanus	502923661	------------G-----A-W-S--NLLAHL		EEV--ERL-EL-RA-L-DIRL-YE--K-R----I
	Meiothermus chliarophilus	654421930	------------G-V---E-W-S--NLLAHL		EEV--ER--ELIRA-LDD-LL-RQ--EIH--V--
	Meiothermus taiwanensis	654415690	--L-------E----R----W-S--GL-ANL		EA-S-K I-AS-EE-RD--RL-RA--LIH----I
	Meiothermus ruber	738244489	--L-------E---------W-S--GL-ANL		EA-S-K I-AS-EE-RD--QL-RT--LIH-----
Other bacteria (0/>500)	*Thermotogales bacterium*	973153189	--V--VP-V-----Y---KQ--SV-N---NI		RD-S-G L----I-GKKSLDM-S--VKL---AD-
	Leptonema illini	488857666	--------V---S-E--IS--DDIDGI-KNL		DAI----L----EE-R---FL-RK-ATIL---DI
	Sphingomonas koreensis	1054815407	--V--V-----------I--F--VDA-L---		PSM--SKM-DN-IEYADMARL-RK-VELAS-V--
	Liberibacter crescens	505086248	-----IP--------L--E---N-DNILLQ-		H-M--SKR-DA-IEHANMARL-RE-VTLR-----
	Acidocella aminolytica	1175533126	--V--VP-----G--A-ID--------L---		PSM--SKR-ES-I-HA-AARL-KQ-VTLRD-A--
	Solibacillus isronensis	1160667273	-----VP-V-E---I---K-H--I--L---M		DS--ASKMKE--V-N--MAHL-KK-ATIH--A--
	Dehalococcoides mccartyi	1155162918	-----VP-------Q--IE---GI-DI-KNL		DKIS-P-LQ---A-NA-VARQ-KI-TTI-C----
	Lysinibacillus saudimassiliensis	674649521	-----VP---E---I---K---SV-NL--HI		DDM--SKMKE--I-NR-QAFM-KE-ATILVEA-I

Figure S10. Partial sequence alignment of a conserved region of DNA polymerase I (PolA) protein showing a 2 amino acid insertion that is specific for all *Deinococcus* group of bacteria.

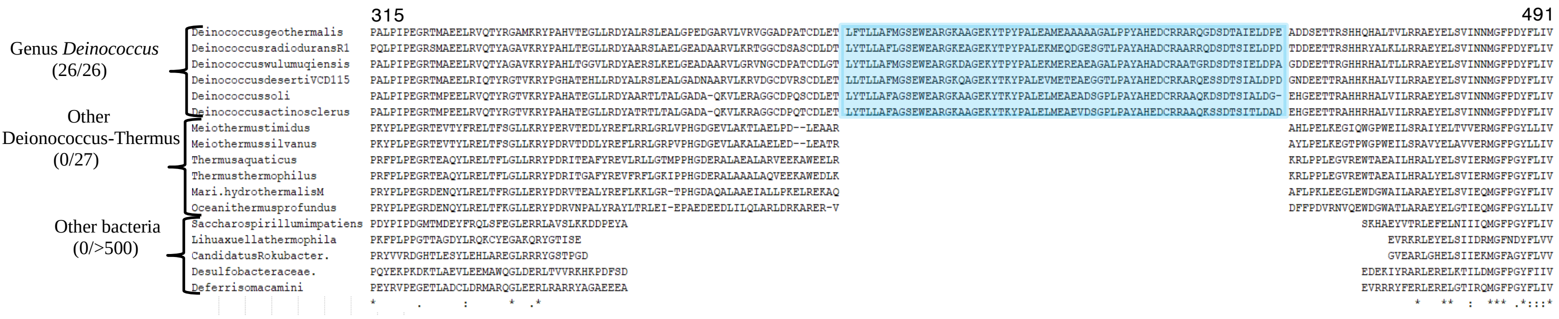

Figure S11. Partial sequence alignment of conserved region of DNA polymerase III alpha (DnaE) protein showing a 65 amino acid insertion that is uniquely shared by all *Deinococcus* group of bacteria.

			75 – 131
Genus *Deinococcus* (24/26)	*Deinococcus geothermalis*	499848670	FYNYASQLGVKPILGYEAYV VP GFGTRRDKKPGVSGEKGIFHLTLLARDFTGYQNL
	Deinococcus murrayi	653254503	-------M------------ -- ---------------------------E-----
	Deinococcus ficus	653266326	-----TGM------------ -- -Q-----RTR-QD---------------E-----
	Deinococcus gobiensis	504498818	-----TGM-----I------ -- -Q-----RTR-QD---------------E-----
	Deinococcus maricopensis	503320977	-----TGM-----I------ -- -V----ERTR-QD---------------E-----
	Deinococcus radiodurans	499189612	-----MGME----I------ -- -------RSRAQD---------------E-----
	Deinococcus actinosclerus	1011240344	-----TGM-----I------ -- -E-----RTRAQD---------------E-----
	Deinococcus grandis	972312411	-----TGM-----I------ -- -E-----RTRAQD---------------E-----
	Deinococcus wulumuqiensis	516483568	-----MGME----I------ -- -M-----RSR-QD---------------E-----
	Deinococcus deserti	502016316	-----QAA-----I------ -- -Q-----RTRAQD---------------E-----
	Deinococcus peraridilitoris	505048928	-----L-M------------ AA-S-F-R---E-----T----------E-----
	Deinococcus pimensis	653296196	-----VSM-----I------ AA-S-H-R---E-----T----------E-----
Other Deionococcus-Thermus (0/27)	*Thermus brockianus*	1103714457	--RK-TE------------- AAES-H-R-R-KGLDG-Y-------K--R-----
	Thermus igniterrae	516808234	--RK-TE------------- AAES-H-R-R-KGLDG-Y-------K--R-----
	Thermus thermophilus	499546976	--KK-TEM-I---------- AAES-F-R-R-KGLDG-Y-------K--------
	Thermus parvatiensis	495906241	--KK-TEM-I---------- AAES-F-R-R-KGLDG-Y-------K--K-----
	Thermus islandicus	551066077	--KK-TEM-I---------- AAES-F-R-R-KGLDG-Y-------K--R-----
	Thermus amyloliquefaciens	740213346	--KK-TSM------------ AAES-Y-R-R-KGLDG-Y-------K--R-----
	Meiothermus chliarophilus	916697201	--K--TEAE----I------ AADS-F-R-Q-KGLDG-Y-------K--E-----
	Meiothermus rufus	1181339566	--K---EM-----I------ AAES-F-R-Q-KGLDG-Y-------QNMQ-----
	Meiothermus timidus	517277716	--K--TEAE----I------ AAES-F-R-Q-KGLDG-Y-------K--E-----
	Meiothermus silvanus	908633755	--K--TEAE----I------ AAES-F-R-Q-KGLDG-Y-------K--E-----
	Meiothermus ruber	738244534	--K--TE------I------ AA-A-F-PEQ-KGLDG-Y-------QNME-----
	Marinithermus hydrothermalis	503468711	--KK-TAA-----I------ AAES-F-R-M-KGLDG-Y----I--EN-------
	Oceanithermus profundus	503223813	--KK-TAA-----I------ AAES-F-RTMRKGLDG-Y-------K--K-----
Other bacteria (0/>500)	*Chloroflexi bacterium*	1125312738	--VA---A-----I-V---I ARQS-F-RD-RIE-HGKP---V---K--------
	Pirellula staleyi	502675500	---K-KA--IN--I-----I AP-S-FE--DAANSKEASY------QNRQ-FK--
	Rhodopirellula baltica	499431723	--RK-KDA-IN--I-----I AP-S-F--GGAS-SKAASY------QNR--FK--
	Opitutaceae bacterium TAV5	918203265	---E-KKA-I--LV-C-L-- AP-S-LE-AGKSEDG-NYY--G----NI------
	Clostridium sp. Marseille-P253	1054809256	--RA-KAA-I-----C-V-- AP-S-F--EA-G---DRYY--V---ENDL--H--
	Opitutaceae bacterium TAV1	494601190	---E-KKA-I--LV-C-L-- AP-S-LE-AGKSEDG-NYY--G----NI------

Figure S12. Partial sequence alignment of conserved region of DNA polymerase III alpha (DnaE) showing a 2 amino acid insertion that is a distinctive characteristics from homologs of *Deinococcus* group of bacteria. Two *Deinococcus* spp. do not contain this CSI.

Group	Species	Accession	101	Insertion	169
Genus *Deinococcus* (25/26)	*Deinococcus deserti*	502015237	SLDNVFDADELREWQEKLARSLNLP	PES	DDFTFTGEIKIDGLSVNLYYLDGTLQWAATRGNGRVGEMVT
	Deinococcus wulumuqiensis	516482604	-------D----D------------	--H	--------L------------G-E-----------------
	Deinococcus radiodurans	499191160	-------D----D---------G--	LDT	--------L--------------E--------------I--
	Deinococcus ficus	653260146	------NDE-----G----------	--Y	--------L------------N-E----------Q---I--
	Deinococcus geothermalis	752549754	------SDE--A--------A----	-DH	-G--Y---L-----------A--V-----------------
	Deinococcus apachensis	518414545	-------DE--A--------A----	-DY	-G--Y---L-----------V--V----------VT-----
	Deinococcus gobiensis	504497482	-------DA----------------	ADY	-S------L-----------KG-E--------------I--
	Deinococcus marmoris	1132142816	-------DA--G--R----------	--H	-E-VL---L-----------R--Q-----------T--I--
	Deinococcus murrayi	653256327	----A-ND------------A-G-L	-DM	----Y---L-V---------V--V----------Q---L--
	Deinococcus swuensis	746725257	------SDA--G--R-----A----	--H	---V----L-----------VN-Q----------S---I--
	Deinococcus grandis	972319274	----A--DA---D-------A-G--	A-H	--------L--------------E----------VT--I--
	Deinococcus actinosclerus	1011239200	----A--DA---D-------A-G--	AD-	--------L--------------E----------VT--I--
	Deinococcus frigens	657675717	----A--DA--D--R----------	--H	-E-ML---L-----------Q--V----------A------
	Deinococcus reticulitermitis	1094409482	------SDH-----G-----A-G--	A-H	---A----L-----------V--E----------A---L--
	Deinococcus aquatilis	517838003	-------DA--G--R----------	ADT	-T------L-----------R--E----------VT--I--
	Deinococcus proteolyticus	503379980	----A--DS-MAG---R---A----	-DH	G--A----L-----------R--E-----------T--K--
	Deinococcus maricopensis	503322439	------NDE--TD-GA----A-GAA	-DT	A-YAY-C-L-----------V--V----------V---I--
	Deinococcus hopiensis	1180562188	----A-SNA--AHFD--V--A----	VGD	QQ-AY-C-L-----------V--V--------D-E---K--
	Deinococcus phoenicis	597706091	----A-SDA--AGFD-RV--A---A	-RE	QH-AY-C-L-----------V--V--------D-E---K--
	Deinococcus pimensis	771654655	-------D---AG---R-S-A-GGG		T---Y-C-L-----------V--E-R--------T---D--
Other Deionococcus-Thermus (0/27)	*Meiothermus silvanus*	502923533	----A-GPEDI-LFE-RI----GM-		AP-PYVL-Y-----------EE---L-G------Q---D--
	Marinithermus hydrothermalis	503469121	----A-NLE-V--FEAR-E-A-GR-		GP-EY-V-Y-----------EE-F-V-G-------T--E--
	Meiothermus timidus	517277018	----A-GQQDIV-FEARV--A-G--		GP-NY-L-Y------I--L-Q--V-V-G------T---D--
	Meiothermus chliarophilus	654421759	----A-GPQDIADFETRA--A-G--		GP-DY-L-Y---------F-Q--L-V-G------T---D--
	Thermus scotoductus	518380761	----A-NF---KAFE-RIG-A-GRE		GP-AY-V-H-V---------E--V-V-G----D-E---E--
	Thermus thermophilus	504442433	----A-NF---KAFE-RIG-A-GRK		GP--Y-V-H-V---------E--V-VYG----D-E---E--
	Thermus parvatiensis	982899939	----A-SFE--KAFE-RIG-A-GRE		GP-AY-V-H-V---------E--V-V-G----D-E---E--
	Thermus brockianus	1103713965	----A-NHE--QAFE-RIE-A-GRK		GP-VY-V-H-V---------EE-V-V-G----D-E---E--
	Oceanithermus profundus	503222790	----A-GP--IAAFE-RV--A-GTE		PPLEYAL-Y------I--L-EG-R-V-G----------E--
Other bacteria (0/>500)	*Tropheryma whipplei*	755157225	------SL-Q--S-Y--TKKICPEG		-QC--VC-L----VG-S-R-AN-Y-IS-----D-AI--DI-
	Streptomyces tsukubensis	750632199	----A--EE--AA-A-RI--DVGT-		--H-LC-L-V---A---T-EK-K-VR-----D-----DI-
	Streptomyces griseoluteus	663177559	----A--DA--AA-S-RV--DVGT-		--HYLC-L-V---A---T-EH-R-TR-----D--A--DI-
	Actinomyces provencensis	1151012413	---D--SIE--KA-EDRVHADTGR-		-LAM-C-A-----A-D-L-V--R--Q-----D-----D--
	Xylanimonas cellulosilytica	502642750	------SV----D-DARV--D-GAG		-VGYLA-V-----AIA-L-EH-R-TR-----D--T--D--
	Rathayibacter tritici	1181002749	------S-E-----C--TVA-ADR-		IAWLT-L-----AIS-H-EQ-R-VT-----D--I--D--
	Propionibacterium acidifaciens	546156869	---D--SV------MARTVEA-GAE		PVWLC-V-----A-D-Q-V--A-AT-----D-----DI-
	Brachybacterium paraconglomeratum	498235416	------SLE-----C-HA-AE-SA-		VR-LN-L-----AI--R-EN-Q-VT-----D--T--D--
	Microbacterium resistens	1055601479	------SVE-----AA-TQAAAGR-		VAWLT-L-----AI--R-E--V-TS-----D-----I--
	Microbacterium indicum	656171461	------SP----A-CVRA-EAAGR-		VRWLT-L-----AI--R-E--V-TS-----D-----I--

Figure S13. Partial sequence alignment of conserved region of NAD (+)-dependent DNA ligase (LigA) protein showing a 3 amino acid insertion that is uniquely shared by the *Deinococcus* group of bacteria. *Deinococcus pimensis* does not contain this insertion.

			265				341
Genus *Deinococcus* (26/26)	*Deinococcus wulumuqiensis*	516481209	NKTNLLQTISAMYKAGKIPDIGALRDESDR	K	EPVRIVIELKRSA	AG	MGTLVLNQLYKYTQLQSSYTVMNLSIVNGEP
	Deinococcus radiodurans	499191008	------------------------------	-	-------------	--	-------------------------------
	Deinococcus reticulitermitis	1094409587	---------------------S--------	-	D-----V----G-		L-----------------F------------
	Deinococcus gobiensis	504497349	---------------------S--------	-	-----------G-		IP---------------T---I---------
	Deinococcus geothermalis	499849419	-----I---------------S--------	-	------V----G-		IP---------------TF------------
	Deinococcus murrayi	653256151	-----I---------------A--------	-	-----------G-		IP--------------TTF------------
	Deinococcus aquatilis	517839262	---------------------S--------	-	-----------G-		IP---------------TF--I---------
	Deinococcus maricopensis	503322881	-----I---------------S--------	-	------V----G-		IP--------------TTF--I---------
	Deinococcus phoenicis	736330843	-----I---------------S--------	-	D-----V----G-		IP---------------TF--I---------
	Deinococcus apachensis	518415676	-----I---------------S--------	-	D----------G-		IP---------------TF--I----M----
	Deinococcus marmoris	1132144081	-----I---------------S--------	-	D----------G-		IP--------------GTF--I---------
	Deinococcus swuensis	746725649	-----I---------------S--------	-	D-----V----G-		IP--------------GTF--I---------
	Deinococcus deserti	502015264	-----I---------------S--------	-	D-----V----G-		IP--------------TT---I-----G---
	Deinococcus actinosclerus	1011238718	-----I---------------A--------	-	D------D---G-		IP---------------TF-II-----H---
	Deinococcus frigens	657674351	-----I---------------S--------	-	D-----V----G-		IP--M-----------GTF--I---------
	Deinococcus peraridilitoris	505047105	-----I---------------AR-T-----	-	-----------G-		IPS--------------TF-II---------
	Deinococcus misasensis	736314419	--S--VS------RQ------S--------	R	-------D---GT		IPE-------------TTF--I--A---K--
Other Deionococcus-Thermus (0/27)	*Truepera radiovictrix*	502943435	---S-I--AAGLVRSKR-E--ANI------		QGM---F----G-		HPE--------------TFS-N--A--DRS-
	Marinithermus hydrothermalis	503469154	--AH-ISQ-ASLV--K--E--A--------		QGL--A-----G-		NPDV-----F-H-N--T-F--NM-A--D---
	Oceanithermus profundus	503222856	--AS-ISQ-ASLVR-K-LE--SG-------		QGM--------G-		NPDV-----F-Q-N--T-F-INM-A------
	Thermus igniterrae	516808786	--AS-IAQ-A-LV--K--E--V--------		QGL--A-----G-		NPQV-------H-A--T-F--NL-A------
	Thermus tengchongensis	740198218	--AG-IAQ-A-LV--K--E--V--------		QGL--A-----G-		NPQV-------H-A--T-F--NL-A------
	Thermus scotoductus	740230202	--AG-IAQ-A-LV--K--E--V--------		QGL--A-----G-		SPQV-------H-A--T-F--NL-A------
	Thermus aquaticus	927054606	--AG-IAQ-A-LV--K--E--V--------		QGL--A-----G-		NPQV-------H-A----F--NL-A--E---
	Thermus thermophilus	499486770	--AS-IAQ-A-LV--K--E--VG-------		QGL--A-----G-		NPQV-------H-A--T-F--NL-A--D---
Other bacteria (0/>500)	*Ignavibacterium album*	504371778	--S--IEK-AELVRE---D--SNI------		DGL--------D-		QP-V-------H--M-VTFG-IM-AL-H-V-
	Clostridium colicanis	1008196356	--AK-IES-A-LV-DK--NG-SD-------		-GM--------D-		NANI-------H-KM-D-FGIIM-AL-----
	Elusimicrobium minutum	501382623	-----IEA-AGLV-DK-VT--ADI------		RGM-L---V--DG		DARV---H---H----T-FS-NM-A--D-R-
	Geoglobus acetivorans	851165112	--A--VEK-AGLARD---EE-KTV------		-GI---V---NG-		NAGV---R--------TTFGII--AL-DNQ-
	Anaerolinea thermophila	973085201	---T-IER-AELVRSDR--E-SD-------		TGM--IL------		QPKR---R---------TFGINM-AL-D---
	Gracilimonas tropica	521071534	--ST-I-K-AGLVNDE--TE-SEV------		-GI----I-----		NAGV----------M-TTFG-I--AL-K-R-
	Rhodohalobacter halophila	1060736857	--AT-I-K-AQLVSDE--T--SEI------		-GM----I-----		NAGV----------M-QTFG-I--AL-Q-R-
	Lachnospiraceae bacterium	1088797531	--A--IKN-ADLV-TK--EG-TD-------		-GM------RHD-		NANI-----F-H--M-DTFG-IM--L-----
	Chloroflexi bacterium	1084617322	---T-IER-AELARK--LD--TD-------		RGMS-IV------		QPRK-------------TFGAQM-AL-E---
	Levilinea saccharolytica	1011525517	---T-IER-ADLVRE-R-DA-SD-------		KGM------R-G-		EPHK---------P---TFG-NM-AL-----

Figure S14. Partial sequence alignment of conserved region of DNA gyrase A (GyrA) protein showing a 1 amino acid insertion that is uniquely shared by all *Deinococcus* group of bacteria. This sequence alignment also contains a 2 amino acid insertion which is only specific for *Deinococcus radiodurans* and *Deinococcus wulumuqiensis*.

Group	Species	Accession	27 … 99
Order *Deinococcales* (27/27)	*Deinococcus deserti*	502015605	YTADDISVLEGMDAVRKRPGMYV Q GGTGIDGYHQLLTEIIDNGIDEGLAGFATEVHVIMHADGSATVTDDGRG
	Deinococcus reticulitermitis	1094409537	-N--Q------------------ - -----------------A---------S---I---E-----------
	Deinococcus grandis	972320201	---A------------------- - ------------------------N----I---L----A-----N---
	Deinococcus actinosclerus	1011238675	---A------------------- - ------------------------N----I---L----A-----N---
	Deinococcus geothermalis	499848956	----S--I-K-LE---------- - -----------------A-------------------------N---
	Deinococcus puniceus	1177843460	---A-----K-LE---------- - -----------------A---------N---------------N---
	Deinococcus radiodurans	499190011	-N--Q------------------ - ---------------------------G-IQIVL-------------
	Deinococcus gobiensis	504497181	----H-TM--------------- - ----V-------------------------RIT-------S-------
	Deinococcus aquatilis	517840584	---A---I-K-LE---------- - -----------------A---------N---------------N---
	Deinococcus wulumuqiensis	516480581	-N--Q------------------ - ---------------------------N-IEIVL----A-----N---
	Deinococcus ficus	916700316	---A--Q---------------- - ----------------------------A-T-VL-----T----N---
	Deinococcus swuensis	746727776	---A-----K-LE---------- - ----V------------A----T----D-I-------------N---
	Deinococcus marmoris	657680678	---A-----K-LE---------- - ----V------------A----T----D-I-------------N---
	Deinococcus apachensis	518414744	-D-SS--I-K-LE---------- - ----V------------A-----G-------------------N---
	Deinococcus maricopensis	503321958	----Q--I-K-LE---------- - ---------------------------DTIQ-----------I-N---
	Deinococcus phoenicis	736328517	-N-SS--I-K-LE---------- - ----V------------A-----G---D---------------N---
	Deinococcus proteolyticus	503380341	----S--I-K-LE---------- - -----------------A---------N-IT-T--E--------N---
	Deinococcus murrayi	653253899	-D-SS--I-K-LE---------- - -----------------A-----G---D---------------N---
	Deinococcus hopiensis	1180559004	-D-SS--I-K-LE---------- - -----------------A-----G---D---------------N---
	Deinococcus pimensis	653297875	-N-SS-T--K-LE---------- - ----V------------A------------T-TF-E--A-----N---
	Deinococcus perariditilitoris	505049945	-N-SS-T--K-LE---------- - -----------M------A-------Y-N--T-TF---A-V----N---
	Deinococcus misasensis	736317881	-N-SN-R----LE---E-----I - --------------V--A---------D--T-TL-K---VS---N---
	Truepera radiovictrix	502942889	----S-K--K-LEG-----A--- - ----V------------A---A---H-DT-E-VLNP------A-N---
Other bacteria (0/>500)	*Enterococcus faecium*	816126482	-D-SQ-Q----LE---------I -S-SSE-L-H-VW--V--S---V------KI---IEK-N-I--I-----
	Juglans regia	1098851128	-GS-Q-Q--Q-L-P--------I -S--PR-L-H-VY--L--AV--AQ----SKID--L-----VSI--N---
	Marinithermus hydrothermalis	503468403	-D-SS-K--K-LEG-----A--I A---T-------S--L--AV--A---Y-N-IK-TL------S-E-N---
	Thermoleophilum album	1093218931	-S-Q--T----LE---------I -S---R-L-H-IY-VV--SV--A----CD-IEIVI-P--AV----N---
	Lactobacillus sharpeae	938894560	-D-SQ-Q----LE---------I -S--VQ-L-H-VW---------A------TIN--VEP-N-I--------
	Clostridium botulinum	409742686	-DESQ-Q----LE---------I -S-SLR-L-H-VY--V--S---A----C-HIE-FI-K-N-I--V-----
	Clostridium butyricum	940832684	-DESQ-Q----LE---------I -S-SLR-L-H-VY--V--S---A----C-HIE-FI-K-N-I--V-----
	Enterococcus faecium	695584429	-D-SQ-Q----LE---------I -S-SSE-L-H-VW--V--S---V------KI---IEK-N-I--I-----
	Emiliania huxleyi	551625470	-G-EQ-T----LEP--------I -S--SR-L-H-VF-VV--SV--A---W----N-TI--S-AIS-S-----
	bacterium F16	1202073469	-SGE--K----LE------S--I -D--EA-F-H-VS-VV--S---A---YC-KIE--INE---LS-Q-----
	Hassallia byssoidea	746979532	-S--Q-Q----LEP--------I -T--PK-L-H-VY-VV--SV--A---YC-H-E-EIN----V--------
	Lactobacillus equigenerosi	949563033	-D-SQ-Q----LE---------I -S-TVQ-L-H-VW--V------A-------IN-TVE--N-I----N---
	Vitrella brassicaformis	873225880	----Q-T----LEP--------I -S--VT---H-VW-VV--AV--A---HCNHI-INL-T---VS-S-N---
	Gossypium hirsutum	1029128219	-NSEQ-Q----L-P--------I -S--PR-L-H-VY--L--AV--AQ-----QID-VL-S---VSI--N---
	Planktothrix agardhii	653002254	-S--Q-Q----LE---------I -S--PR-L-H-VY-VV--A---A---YC-HIEIDFN-N--V--------

Figure S15. Partial sequence alignment of conserved region of DNA gyrase subunit B (GyrB) protein showing a 1 amino acid insertion that is specific for all *Deinococcus* group of bacteria.

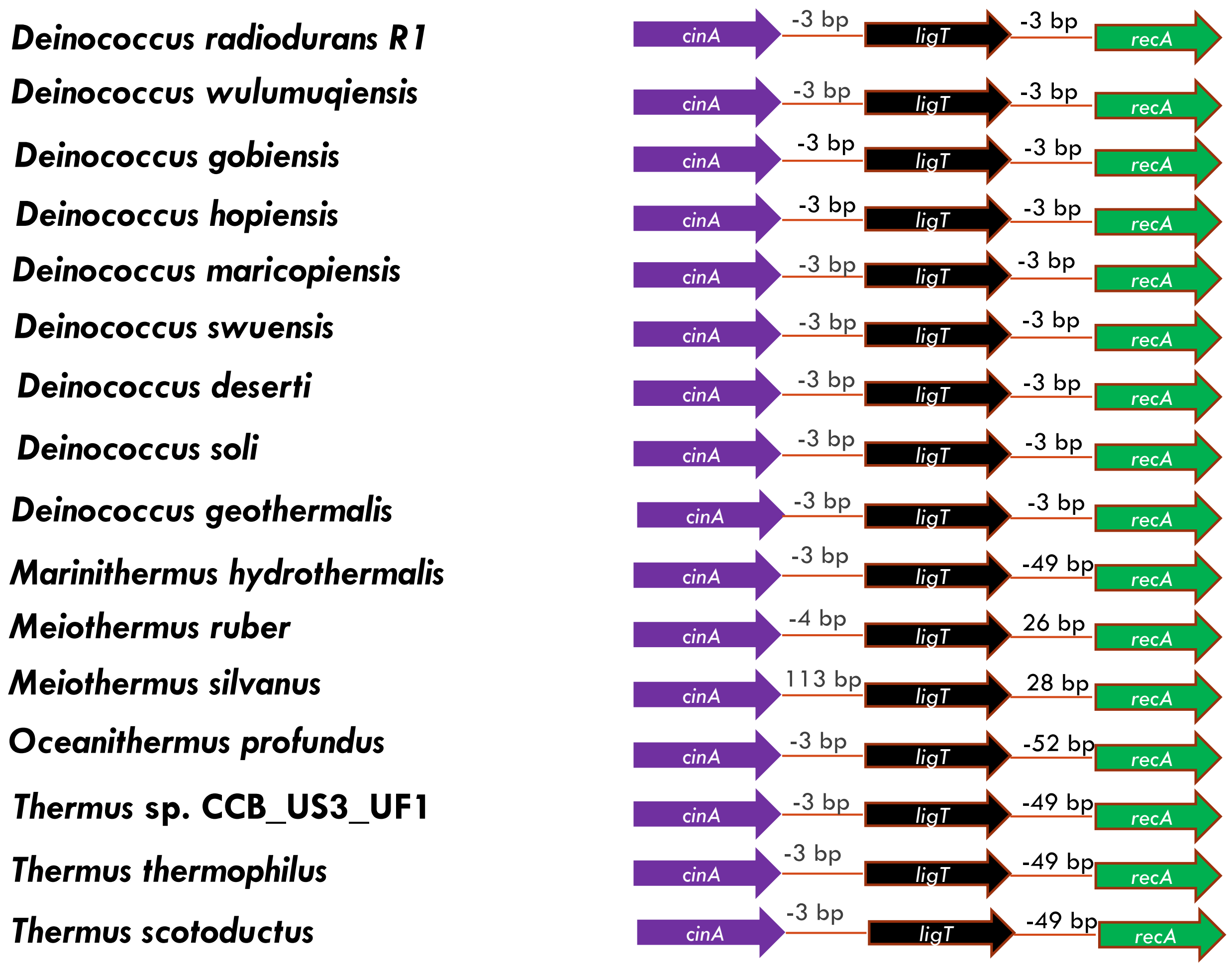

Figure S16. Diagrammatic representation of genomic neighborhood analysis result of DNA recombination protein RecA for representative Deinococcus-Thermus spp. Gene of RecA protein is present in a genetic linkage with genes of LigT and CinA proteins throughout all Deinococcus-Thermus spp. The intergenic distance among them is less than 200 bp and is present in the same direction. Genes of RecA, LigT and CinA proteins should be present in same operon among Deinococcus-Thermus phylum.

(A)

```
                                109        CXXC       131 175       CXXC      191 197              CXXC          229
Deinococcus radiodurans         RLLYARVGTPYCPICGRKIEKQ FSFNSPYGACGDCAGIG SEFMRERLEELMELRPCPTCGGTRYKPEILAV
Deinococcus maricopensis        RLLYARVGTPYCPICGRKIERQ FSFNSPYGACGDCAGLG SEYMREKLEELMELQPCPTCGGTRYKPEILAV
Deinococcus gobiensis           RLLYARVGTPYCPICGRKIEKQ FSFNNPYGACPDCAGLG SDFMREKLEELMELRPCPTCGGTRYKPEILAV
Deinococcus phoenicis           RLLYARVGTPYCPICGRKIEKQ FSFNNPYGACPDCAGLG SEFMREKLEELMELRPCPTCGGTRYKPEILAV
Deinococcus geothermalis        RLLYARVGTPYCPICGRKIEKQ FSFNSPYGACSDCAGLG SDFMREKLEELMELRPCPTCGGTRYKPEILAV
Deinococcus deserti             RLLYARVGTPYCPVCGRKIEKQ FSFNSPYGACGDCAGLG SEFMREKLEELMELQPCPTCGGTRYKPEILAV
Deinococcus grandis             RLLYARVGTPYCPVCGRKIEKQ FSFNSPYGACGDCAGLG SDFMREKLEELMELQPCPTCGGTRYKPEILAV
Mycobacterium sp.1465703.0      RLLYARAGTPHCPICGERIARQ FSFNSPYGACPECSGLG SEQMKERYEGFMRDVPCPVCEGTRLKPEILAV
Modestobacter marinus           RLLYARAGQPHCPNCGKPISRQ FSFNSPFGACPECTGIG SEFMKDKYEGYMRDVPCPVCHGTRLKPEILAV
Eggerthella lenta               RLLFARVGVPHCPECGRVIKKQ FSFNAPYGACPDCLGIG SDAQREKLASYFAIVPCPTCGGKRLKPEILAV
Meiothermus timidus             RLLFARVGTAFCPHCGRPIERQ FSFNAPYGACPDCSGLG SEGLREALEAYMTLQPCPACSGTRYKKEVLSV
Meiothermus cerbereus           RLLFARVGTAYCPHCGRPIERQ FSFNAPYGACPDCSGLG SESLREALEAYMTLKACPACGGTRYKREVLSV
Meiothermus ruber               RLLFARVGTAYCPHCGRPIERQ FSFNAPYGACPDCSGLG SEGLRETLEAYMTLKACPACGGTRYKREVLSV
                                ***:**.*  .** **. * :* **** *:*** :* *:* *:  ::     :    **.* *.* * *:*:*
```

The other two CXXC motifs in this protein are shown in Figure 8

(B)

```
                                    228                  CXXC                  272
                                                                               CXXC
Deinococcus radiodurans             DGEGGGFQHSHHVYGRAGQPCDRCGTPIEKIVLGQRGTHFCPVCQ
Deinococcus misasensis              DGNPAYFQFEHMAYAREGEPCQRCQQPIAKYWLAQRGTHHCPNCQ
Deinococcus perariditoris           NGESGWFQFRHNVYARKGKACARCGGTIEKIVLGQRGTHFCPECQ
Deinococcusmaricopensis             DGLSGLFQQQHNAYARDGEPCARCGTTITKSVLAQRGTHHCPACQ
Deinococcus geothermalis            DGAVGFFQGQHAVYGRTGQLCPRCGTPIQKMVLAQRGTHFCPACQ
Deinococcus frigens                 DGVSGLFQHEHHVYGKGGQPCPRCGTDIVKTVLAQRGTHFCPKCQ
Deinococcus deserti                 DGVSGLFQFSHRAYAREGQPCERCGTSIEKIVLGQRGTHFCPQCQ
Deinococcus soliChaetal.2016        DGLSGLFQHAHNVYGKGGEPCPRCGTPIEKSVVAQRGTHHCPQCQ
Deinococcus gobiensis               DGEQGGFQGRHQAYGRAGQPCARCGTPIAKIVLGQRGTHFCPQCQ
Deinococcus reticulitermitis        DGEPGGFQHAHRVYGKAGEPCARCGTPIQKVVLGQRGTHFCPQCQ
Deinococcus wulumuqiensis           DGEGGGYQTRHHAYGKTGQPCERCGTPIEKIVLGQRGTHFCPACQ
Marinithermus hydrothermalisDSM     DGALGRFQVQHKVYGRPGAPCVRCGTPILKAVVAGRGTHFCPRCQ
Thermus thermophile                 DGLPGGFQTRHAVYGREGLPCPACGRPVERRVVAGRGTHFCPTCQ
Thermus scotoductus                 DGLPGSFQMRHAVYGRTGLPCPVCGTPIAKRVVAGRGTHFCPRCQ
Morganel lamorganii                 DGKPGYFAQELFVYGKQGECCANCGQKIEVVKVGQRSTFFCPACQ
Streptococcus parasanguinisATCC     FGEDGTMQEEHQVYGKTGQPCLRCGTPIEKIQLGGRGTHFCPHCQ
Carnobacterium pleistocenium        LGEAGTFQMKLAVYGKKGIPCIRCGTPIEKIKVAQRGTHFCSNCQ
Enterococcus canis                  LGEAGKFQLSLNVYGQTGNPCPRCGTPIQKIKVAQRGTHFCPNCQ
Enterococcus gallinarum             LGEAGHFQVSLHVYGQTGNPCVRCGTPIVKTKVAQRGTHYCPFCQ
                                     *  .       .* : *  *  *   :    :. *.*. *  **
```

(C)

```
                                        190                CXXC              229
Deinococcus radiodurans                 NEAVMDLGATICVPKSPACDRCPVSAHCAAYQLGQPGDFP
Deinococcus deserti VCD115              NEALMDLGATICTPRSPRCSDCPVSKYCCAFAEGRPAAYP
Deinococcus wulumuqiensis               NEAVMDLGATVCVPKSPACDRCPVSAHCAAYRLGRPSDFP
Deinococcus hopiensis                   NEAVMDLGATVCTPKVPKCPDCPVSLWCAAFQSGQPAAYP
Deinococcus geothermalis DSM1130        NEALMDLGATVCTPKAPQCGECPLRRWCAAFQLGDPAAFP
Deinococcus phoenicis                   NEAVMDLGATVCTPKAPKCGACPVSAWCAALGSGEPAAFP
Deinococcus apachensis                  NEAVMDLGATICTPRAPKCGECPVRAWCAAFASGHPAAFP
Deinococcus aquatilis                   NEAVMDLGATICTPKAPRCAVCPLAAWCEARASGQPTAFP
Deinococcus marmoris                    NEAVMDLGATVCTPKAPRCAECPLSAHCAAFKRGTPTAFP
Deinococcus swuensis                    NEAVMDLGATVCTPRAPRCPECPLAARCAARASGTPAAFP
Thermus thermophilus                    NQALMELGATVCLPKRPRCGACPLGAFCRG--KEAPGRYP
Meiothermus rufus                       NQALMELGATVCTPRKPNCPGCPLVTFCRG--QGQPERYP
Meiothermus ruber DSM1279               NQALMELGATVCTPQNPGCGGCPVAAFCQG--KASPAHYP
Meiothermus taiwanensis                 NQALMELGATICTPQNPGCGGCPVAAFCRG--KASPGRYP
Meiothermus timidus                     NQAMMELGATLCTPQKPACTSCPLARWCAG--QASPERYP
Lentisphaeria bacterium TMED266         NEALMELGATVCLPQNPSCDVCPLSTACQAKLLDDVSRFP
Nitrospina gracilis 3/211               NQSLMELGATLCLPQNPMCLLCPIVQHCEAHRQGEPEKFP
Rhodothermus marinus SG0.5JP17-1        NQALMELGATVCTPVQPRCSACPLRRACRAWAMGDPTAFP
Ardenticatena maritima                  NQALMDVGAEICTPRSPRCLLCPVQTHCAAAAHQRQHDLP
Synechococcus sp.PCC8807                NQALMDLGATLCTAKTPACPRCPWQNHCTAYLKHQPTDFP
Geitlerinema sp.PCC7105                 NQAFMDLGATLCTPQNPACLLCPWRESCQAYNLDLQSELP
Truepera radiovictrix DSM17093          NEALMELGALVCTARAPQCPRCPVQAHCGAYQQGAVARFP
                                        *::.*::**  *    * *  **    * .         *
```

(D)

```
                                   187                                                               CXXC 254
Deinococcus radioduransR1          PVDGNMERAAKRLELVPAAWNSHKVERWYAEVMPADWETRFALHISGVRHGRDTCRSKHPLCPQCPLR
Deinococcus geothermalis           PIENNIHRVAGRLDLFPSRWNVLKAERWFDEVLPRDWLDRATFHVSAIRHGRQTCRAQRPRCACCVLQ
Deinococcus marmoris               PVDTHIHRIARRLELVPEAWNAVKVERWFDEVLPREWAARYTFHVSAIRHGRQTCRARNPACGVCVLR
Deinococcus puniceus               PVDTHIGRIAARLEWVPARWNAIKVERWFDEVLPRDWASRYGFHVSAIRHGRETCRAQRPDCEACVLR
Deinococcus aquatilis              PVDTHIGRIAARLEWVPARWNAIKVERWFDEVLPRDWPSRYGFHVSAIRHGRETCRAQRPLCGECVLR
'Deinococcus soli'Chaetal.2014     PVEGNLDRLARRLEWVPDTWTGARVERWFDATVPRTTPDRLRLHVAGVRHGREVCLSRHPSCDACVLA
Deinococcus reticulitermitis       PVDGNMERAAKRLELVPAGWSSDRVERWYDEAAPRDWETRFALHLSGVRHGRVTCRPQRPLCGECVLR
Deinococcus wulumuqiensis          PVDGNMERAAKRLELVPAAWNSHKVERWYAEVVPGDWETRFALHISGVRHGRDTCRSKHPLCPACPLR
Deinococcus ficus                  PVDGNIERTLKRLEFVPPNWSAERTERWFDRVVSREWALRAALHVAGVRHGRHTCLPRNPRCPACPLL
Deinococcusd eserti                PVDTNIERIAKRLELVPQRWTPEKVERWFDAVVRRDWAERATFHVAGVRHGRLTCRPRDPRCDQCVLR
Aminomonas paucivorans             PVDTHVARICRRLGWVPANLPPHRIQRVMEETVARER--FQGAHLNLIAHGRAVCRARSPRCPACVLV
Aminobacterium colombiense         PVDTHIHRFSKRIGWAHDRCKPEEIEGMLEQVVPEER--YLGGHINIITHGRNICLARQPRCDKCSVN
Acetomicrobium thermoterrenum      PVDTHVNRLCKRIGWVSPKSTPEETQKIMGSVIPSDL--YWSAHLDIISHGRNICVSRRPKCTICPLN
Actinobacteria bacterium           PVDTHVYRVGKRLGIIPTKASVEKAHALMDRLVPDDI--KYRLHVNLVDHGRRICVARKPKCPICCLN
Dehalococcoidia bacterium          PVDTHVYRVARRLGLVPPRVGAAEAHGLLESMLTPDE--VYPFHMSLIKHGRRICKAQRPLCPRCVLA
Chloroflexi bacterium              PVDTHVFRVARRLGLLHSKVSAEQAHEILENLIPAGQ--VYEFHLNMVEHGRKVCKAQRPRCHLCVLR
                                          *:: ::     *   *:          . .     *:  : ***  *  : * *  *
```

(E)

```
                                  734                                773
                                  CXXC                          CXXC
Deinococcus radiodurans           CLCCGKRT----QLQVDHIQSRYAGGTHDLDNLQLLCQVCNNLK
Deinococcus frigens               CLCCGSTT----RLQVDHIQPRYLGGSHDTENLQTLCGICNRLK
Phormidium ambiguum IAMM-71       CLCCGEDS--RNVLVIDHIIPRYHGGTHSLDNLQTLCRKCNNLK
Methanobacterium congolense       CLCCGEEK--KKLLEVDHINPRYFGGNNSIDNLQTLCRYCNTTK
Methanobacterium formicicum       CLCCGEDH--KQILEVDHVNPRYMGGKDSIENLQTLCIYCNTAK
Methanosaeta harundinacea         CLCCGTTK--RSFLQVDHIDPRYLGGSNNPENLQTLCGECNNKK
Chondromyces crocatus             CLACGSNR----TLQVDHIIPVYHAGSHEPDNLQTLCKRCNGLK
Sorangium cellulosum              CLACGTTR----NLNADHVVAVYVGGSNEVGNLQTLCRQCNILK
Pseudanabaena biceps PCC7429      CLCCGDH----RRLQVDHVFPFILGGQTSIDNSQTLCMRCNGFK
Desulfosporosinus sp.Tol-M        CRCCGRELGKGIKLEIDHIVPVKMGGQTTLENLQILCKTCNMEK
                                   *. **          :**:     .*     * ***  **  *
```

(F)

```
                                       137            CXXC                 CXXC  182
Deinococcus radioduransR1              SYKLLGLAGVIPQTARCARCGAPDPE----HPDPLGGQLLCSKCAALPPY
Deinococcus deserti VCD115             SYKLLGLAGMVPQTARCARCGQPDPA----HPDPLAGQLLCATCAALPAY
Deinococcus gobiensis I-0              SYKLLGLAGIVPQTARCARCGEDHPA----HPDPLGGQLLCGTCAALPPY
Deinococcus aquatilis                  GFKLLGLAGFVAQTARCARCGADQPA----HPDPLAGQLLCGNCASLPAY
Deinococcus apachensis                 SYKLLGLAGFVLQTARCARCGAPDPA----YPDPLGGQLLCGNCSSLPAY
Deinococcus geothermalis DSM1130       SYKLLGLAGFVPQTGRCARCGAAAPT----HPDPLGGQLLCGACASLPAY
Deinococcu sphoenicis                  SYKLLGLAGFVPQTARCARCGTADPA----HPDPLGGQLLCVACASLPAY
Amycolatopsis pretoriensis             FLRAMSYEGWAPALTECARCGLPGPH---VAFSVPAGGSMCQDCRVPGSV
Streptomyces sp.AA4                    FLRAMAYAGWAPAITECARCGLPGPH---AAFNVAAGGSMCPDCRIAGSV
Amycolatopsis decaplanina              FLRAMAYAGWAPAITECARCGLPGPH---KAFSVSAGGSMCPDCRVPGCV
Herbidospora mongoliensis              FLRSLAVAGYAPALSECARCGAEAV----RAFAIVAGGVVCGTCRPSGAA
Nocardioides dokdonensis FR1436        LLRSLSVAGYAPSFDHCARCGEQGPH---RWFNPSMGGMLCSTCRLPGSA
Truepera radiovictrix DSM17093          GWRLLAQGGLAPRLARCARCGGPLEGSGEGRFDVAAGGLSCAACASGFRV
Deinococcus proteolyticus              SYKLLALAGFPQRTRMCARCGAADPQ----HPDPFGGELLCGRCSHQRAL
Deinococcus wulumuqiensis              SYKLLGLAGVIPQTARCARCGAPEPQ----HPDPLGGQLLCGKCAALPPH
                                         : :   *       *  *              *    *  *
```

(G)

```
                                     23                                 CXXC      CXXC                               109
Deinococcus radiodurans              KSAQRLAFHLFEQPREDIERLASALLEAKRDLHVCPICFNITDAEKCDVCADPSRDQRTICVVEEPGDVIALERSGEYRGLYHVLHG
Deinococcus wulumuqiensis            KSAQRLAFHLFEQPREDIERLASALLEAKRDLHVCPICFNITDAEKCDVCADTSRDQQTICVVEEPGDVIAIERSGEYRGLYHVLHG
Deinococcus frigens                  KSAQRLAFYLFEQPREDIERLAGSLLSAKRDLHSCPICFNITDAERCDVCSDPARDQAIICVVEEPGDVIAIERSGEYRGLYHVLHG
Deinococcus geothermalis DSM1130     KSAQRLAFYLFEQPREDIERLAGAILEAKRDLHTCPVCFNITDAERCDVCSDPTRDQSVICVVEEPGDVIAIERSGEYRGLYHVLHG
Deinococcus gobiensis I-0            KSAQRLAFHLFEQPREDIERLASALLSAKRDLHTCPICFNITDAETCDVCSDPSRDQNLIAVVEEPGDVIAIERSGEYRGLYHVLHG
Deinococcus murrayi                  KSAQRLAFHLFEQPREDIERLAGALLAAKSELHTCPVCFNITDAERCDVCSDPSRDQGVICVVEEPGDVIAIERSGEYRGLYHVLHG
Deinococcus apachensis               KSAQRLAFHLFEQPREDIERLAGALLEAKRDLHTCPICFNITDAERCDVCSDPSRDQNMICVVEEPGDVIAIERSGEYRGLYHVLHG
Deinococcus actinosclerus            KSAQRLAFHLFEQPREDIERLSRALLEAKRDLHTCPVCFNITDAERCDVCSDPSRDQGVICVVEEPGDVIAIERSGEYRGLYHVLHG
Deinococcus grandis                  KSAQRLAFHLFEQPREDIERLSRALLEAKRDLHTCPVCFNITDAERCDVCSDPSRDQGVICVVEEPGDVIAIERSGEYRGLYHVLHG
Butyricicoccus desmolans             KTAQRLAFHVLDLPKEDAERFADAIREAKARTFTCKRCQNLTDTETCPICADKSRDQKTICVVAEPRDVIAFERTKEYKGLYHVLHG
Cald anaerobius fijiensis DSM1791    KTAQRLAFYLLNSPREYVESLARAMVEAKNKLKYCSVCMNITDSDVCSICSDPVRDKSTICVVEDPRDVVAMEKTKNYKGLYHVLHG
Alkaliphilus transvaalensis          KTAQRLAFHVISLSQEEADQLSSAIISAKRNVKYCEICTNLTDMDTCSICKDKSRDASSICVVEDPRDVVAMEKTKEFRGFYHVLHG
Defluviitalea phaphyphila            KTAQRLAFHIINMPDENVENLSSAIMEAKKNIKYCSICCTLTDKEKCDICSDPKRDHKTIMVVEDPRDLVAYEKTKEYKGLYHVLHG
Thermoanaerobacterium thermosac      KTAQRLAFYILDMPKDDVINLSNAILEAKNNLKYCNKCYNFTDSDLCNICSDETRDSSTICVVSDPKDVVAMEKTREYRGLYHVLHG
Caldanaerobacter subterraneus        KTAQRLAFFIINMPLDEVRSLSQAIIEAKEKLRYCKICFNIADKEVCDICSDENRDHSTICVVSHPMDVVAMEKVKEYKGVYHVLHG
Thermoanaerobacter kivui             KTAQRLAFFIINMPLEEVKSLSQAIIEAKEKIRYCKICYNITDTEICNICSDKERDHSLICVVSHPMDVVAMEKTREYKGVYHVLHG
Ruminococcus callidus ATCC27760      KSAQRLAYSIISRPEEDVERFANALLSAKRDIHYCPCCQNLTEMELCSVCADEERDHSLICVVESPKDVTALERTGEYTGVYHVLHG
Truepera radiovictrix DSM17093       KTAQRLAFYLFNQPEEDVKSLAEALLNAKGNLRQCERCFNITDQEVCSVCRDPGREQGLICVVEAPADLLAIERSGEYSGLYHVLHG
                                     *:*****: ::.   :    :: ::  **     *  * .::: : * :* *  *:   * **  * *: * *:  :: *.******
```

(H)

```
                                  395   CXXC                                   442
Deinococcus radiodurans           SEPCGNCDVCLNPPRVRDLTREAQMALSATIRTGNRFGAAHLTDVLLG
Deinococcus wulumuqiensis         PGPCGNCDLCHTPPQVRDLTREAQMALSAAIRTGNRFGAAHLTDVLLG
Deinococcus actinosclerus         HGPCGNCDTCLNPPQVRDMTREAQMALSAAIRTGNRFGAAHLTDVLLG
Deinococcus deserti VCD115        REPCGNCDVCLSPPRVQDATREAQMALSAAIRTGNRFGAAHLTDVLLG
Deinococcus swuensis              DEPCGNCDICLNPPRVRDATREAQMALSAAIRTGNRFGAAHLTDVLLG
Deinococcus marmoris              NEPCGNCDICLNPPRVRDATREAQMALSAAIRTGNRFGAAHLTDILLG
Deinococcus murrayi               PHPCGNCDVCLAPPRVRDATREAQMALSAAIRTGNRFGAAHLTDVLLG
Deinococcus phoenicis             AEPCGNCDVCLNPPRVRDATREAQMALSAAIRTGNRFGAAHLTDVLLG
Deinococcus apachensis            EKPCGNCDVCLNPPRVRDATREAQMALSAAVRTGNRFGAAHLTDVLLG
Rhodanobacter sp. Soil772         PGPCGHCDNCVAPPKTWDATVPAQKALSAVYRTGQRFGSGHVIDVLRG
Nitrosomonas nitrosa              PGRCGQCDNCLNPVDTWDATEAARMALSCVYRTGQRFGAGHLIDVLLG
Haliea salexigens                 PQACGNCDTCLEPVPTWDGTEAARMALSAVYRTGQRFGVNHLIDVLRG
Methylococcus capsulatusstr.Bat   ERPCGNCDNCLNPVQTWDATEAARKALSCVYRSGQRFGAHYVIDLLLG
Methylobacter luteus              AQPCGNCDTCLEPVVTWDGTLAAQQALSCIYRTEQRFGVNYLIDVLLG
Thioalkalivibrio sulfidiphilus    PEPCGNCDTCLEPPETWDATVAAQKALSCVHRTGQRFGVNHVLDVLLG
Sulfurifustis variabilis          PRPCGNCDTCLDPPETWDATVPAQKALSCVHRTGQRFGVSYLVDVLLG
Thermithiobacillus tepidarius     PAPCGNCDTCLEPVETWDGTEAAQMALSCVYRTGQRFGVTHLIEVLQG
                                     **:** *  *  . * .  *:  **.  *: :***  :: ::* *
```

(I)

```
                                  450               CXXC        483
Deinococcus radiodurans           RMEAGLASYRSHLHEGEPCPLCLQTVHEVPEGES
Deinococcus grandis               RLEAGLASYRAHLHVGDDCPLCGQTVTVLPDAPR
Deinococcus deserti VCD115        RLEAGLASHRAHLHVGDDCPLCGQTVRQLPSAPV
Deinococcus ficus                 RLEAGLASHRAHLHVGDDCPLCGQTVQRLPNAPV
Deinococcus gobiensis             QIDAGVAAYRTHLHVGEPCPLCAQTVRTLPDTPP
Deinococcus geothermalis DSM1130  RLTAGLSAYRAHLHVGEPCPLCEQTVRVLPQASA
Deinococcus murrayi               RARAGLAAYRSHLHLGEPCPLCGGAVQTLPPPAE
Meiothermus cerbereus             KLQQGLGLYHAHLKQGEPCPLCGHPVENLPPPKP
Meiothermus ruber                 KLRQGIAHYHPHLKVGEPCPLCGHPVAALPPAQP
Meiothermus taiwanensis           KLRQGIAHYHPHLKVGEPCPLCGHPVAALPPAQP
Thermus amyloliquefaciens         ERRLGLLAYRDLLRPGEPCPLCGGVVHGLPPAPA
Thermus tengchongensis            ERRLGLLAYRDLLRLGEPCPLCGSVVHGLPPVLE
Thermus arciformis                ERRLGLLAYHDLLRPGEPCPLCGGVVHRVPERPS
Thermus oshimai                   ERRQGVLAYRDLLQVGAPCPLCGGVVHALPEVPQ
                                  .   *:  :   *: *  ****   *   *
```

(J)

```
                                  3      CXXC          CXXC            41
Deinococcus radiodurans           KVKTNYICNSCGYQSAKPLGRCPNCQAWNSFEEEVPTAS
Deinococcus wulumuqiensis         KLKTNYVCTSCGYQSAKPLGRCPNCQAWNSFEEEVPTAS
Deinococcus marmoris              RVTTKYVCNSCGYTSAKPLGRCPNCQAWNSFEEEVPSVT
Deinococcus frigens               RVTTKYVCNSCGYTSAKPLGRCPNCQAWNSFEEEVPSVT
Deinococcus deserti VCD115        KVRVGYVCNSCGYQSAKPLGRCPNCQAWNSFEEETPAVA
Truepera radiovictrix DSM17093    KVGTTYVCSECGTHSPVKMGRCPRCGTWGSMAAQAPAPA
Meiothermus Silvanus DSM9946      RTSTQYRCTACGYKSVKALGRCPNCGAWDSFKEEAPEAP
Meiothermus rufus                 RASAQYRCVECGYQSVKPLGRCPGCGAWESLKEVAEARP
Meiothermus cerbereus             RASIQYRCIECGYKSVKELGRCPNCGAWDSFKEEAPPPK
Oceanithermus profundus DSM14977  KPKSQYRCVECGYRAPKSLGRCPGCGAWGSFLEERSDGG
Marinithermus hydrothermalis DSM  MKKADYRCVECGYVTPKPLGRCPGCGAWDSFQRTVPDSP
Thermus filiformis                MAKAQYRCVECGYRTPKPLGRCPGCGAWNSFKEEAPSPP
Thermus scotoductus               MAKTSYTCVECGYRTPKPLGRCPACGAWESFQEVAPSPR
Lachnospiraceae bacterium JC7     KITSKFYCKECGYESAKWLGQCPGCRAWNSFVEEPVAST
Oribacterium sp.C9                KITSKFFCKECGYESAKWLGQCPGCRAWNSFVEEPVAGT
Butyrivibrio sp.WCD2001           KIKTVFFCQSCGYESAKWMGQCPGCKEWNSFVEETIKPS
Lachnospiraceae bacterium P6A3    KQTSVYFCQSCGYESAKWMGQCPGCHEWNTFVEEPVVKA
                                       : *  **  :    *:** *  * ::
```

(K)

```
                                       388                       CXXC                     447
Deinococcus radiodurans                IPQIMRVLPDKRPADAAPFEFPTHCPVCGHEAVRAEGDANTYCPNPACPAQSFERIRYFV
Deinococcus deserti VCD115             IPQIMRVVTEKRPEKTTPFVFPTYCPECGHEVTRAEGDANTYCPNPACPAQRFERIRYFV
Deinococcus aquatilis                  IPQIMRVLVEKRPEGTQPYAFPTHCPQCGHEAVRTEGDANTYCPNPACPAQQFERIRYFV
Deinococcus geothermalis               IPQIMRVVLEKRPPDAAPYRFPTHCPECGHEVVRAEGDANTYCPNPACPAQQFERLRYFV
Deinococcus grandis                    IPQIMRVLTEKRPADAQPFEFPTHCPECVHEVTRAEGDANTYCPNPACPSQAFERIRYFV
Deinococcus soli Chaetal.2016          IPQIMRVLTDRRPDGAQPFEFPTHCPECGHEVTRAEGDANTYCPNPACPSQAFERIRYFV
Deinococcus gobiensisI -0              IPQIMRVLPEKRPEGTVPFAFPEHCPVCGHGAVRAEGDANTYCPNPACPAQNFERIRYFV
Deinococcus reticulitermitis           IPQIMRVILDKRPEGAEPFAFPTHCPVCGHEATRAEGDANTYCPNPACPAQRFERIRYFV
Deinococcus wulumuqiensis              IPQIMGVVLDRRPADAAPFEFPTHCPVCGHVAVRAEGDANTYCPNPACPAQSFERLRYFV
Truepera radiovictrix DSM17093         IPEVIRVLTEARTAELPPYVFPETCPACGEALIE--DGANVRCVNLACPAQVLARLSHYA
Meiothermus ruber H328                 IPEVLRVVTEAPRGH-APVEWPTHCPECGTALEL--SGKIHLCPNPLCPAKAFEAIRHFA
Meiothermus cerbereus                  IPEVLRVVTEAPRGH-EPVEWPSHCPECGVELLL--SGKIHLCPNPLCPAKAFESIRHFA
Oceanithermus profundus DSM14977       IPEILRVIKEKRTGKEEPIVFPYHCPECGANLVE--DGKIHRCPNPLCPAKAFQQLRHWA
Marinithermus hydrothermalis DSM       IPEVLRVLKEERTGTERPLVFPDACPACGHALVL--EGKIHRCPNPLCPAQAFERIRHYA
Thermus filiformis                     IPEVLRVLKERRTGKERPIRWPEACPECGHRLVK--EGKVHRCPNPLCPAKRFEAIRHYA
Thermus igniterrae                     IPEVLRVLKERRTGEERPIRWPERCPECGFRLVQ--EGKVHRCPNPLCPAKRFEAIRHYA
Thermus oshimai JL-2                   IPEVLRVLKERRTGAERPIRWPETCPECGHALVK--EGKVHRCPNPLCPAKRFEAIRHYA
Thermus scotoductus                    IPEVLRVLKEKRTGEERPIRWPETCPECGHRLVK--EGKVHRCPNPLCPAKRFEAIRHYA
Thermus thermophiles JL-18             IPEILRVLKERRTGEERPVRWPETCPECGHRLLK--EGKVHRCPNPLCPAKRFEAIRHFA
                                       **:::  : :       *  :*  ** *         .    * *  **::    : ::.
```

(L)

```
                                         58           CXXC                                   115
Deinococcus radiodurans                  MTANCTG-PAPKPCGECESCLAVRAGSHPDVMEIDAASNNSVDDVRDLREKVGLAAMRG
Deinococcus swuensis                     MTANCTG-PAPKPCGECDSCLSVRAGSHPDVMEIDAASNNSVDDVRDLRELVGLAAMRG
Deinococcus frigens                      MTANCTG-PAPKPCGECESCLSVRAGSHPDVMEIDAASNNSVDDVRDLRELVGLAAMRG
Deinococcus geothermalis DSM1130         MTANCTG-PSPKPCGECESCLAVRNGSHPDVLEIDAASNNSVDDVRDLREKVGLAAMRG
Deinococcus sp.Leaf326                   MTANCQS-EGPKPCGECESCLAVRAGSHPDVMEIDAASNNSVDDVRDLREKVGLAAMRG
Deinococcus reticulitermitis             MTANCSA-PGPKPCGECESCLSVRAGSHPDVLEIDAASNNGVDDVRDLREKVGLAAMRG
Deinococcus puniceus                     MTANCTG-PLPKPCGECESCLSVRSGNHPDVLEIDAASNNSVDDVRDLREKVGLAAMRG
Deinococcus deserti VCD115               MTANCSG-PLPKPCGECESCLSVRAGSHPDVLEIDAASNNSVDDVRDLREKVSLAAMRG
Oceanithermus profundus DSM14977         MSVGCAAEPENRPCGECENCRMVREDRHPDVVEIDAASNNSVEDVRELRERILLAPLVA
Truepera radiovictrix DSM17093           MAVNCEG-E-DRPCGACESCLLVRRGAHPDVTELDAASNNSVEDIRDLREKVRLASLRG
                                          *: .*       **. *  *    .   *: *:*.***..::::*::*: :  :
```

Figure S17. Sequence alignment showing the presence of CXXC motifs (highlighted) in different DNA Repair proteins. (A) UvrA protein; (B) MutM protein; (C) MutY protein; (D) Nth protein; (E) Rad25 protein; (F) RecO protein; (G) RecR protein; (H) RecQ protein; (I) SbcC protein; (J) RadA protein; (K) LigA protein; (L) DnaX
